# Supplementary material for: A unified model library maps how neuromodulation reshapes the excitability landscape of neurons across the brain
Source: PLoS Comput Biol. 2025 Dec 1;21(12):e1013765. doi: 10.1371/journal.pcbi.1013765 (PMC12680334; doi:10.1371/journal.pcbi.1013765)
Supplement: S1 Fig — Comparing NEST, Brian 2 and our implementation when using models from [14]. (PDF) [file pcbi.1013765.s001.pdf]

Supporting information

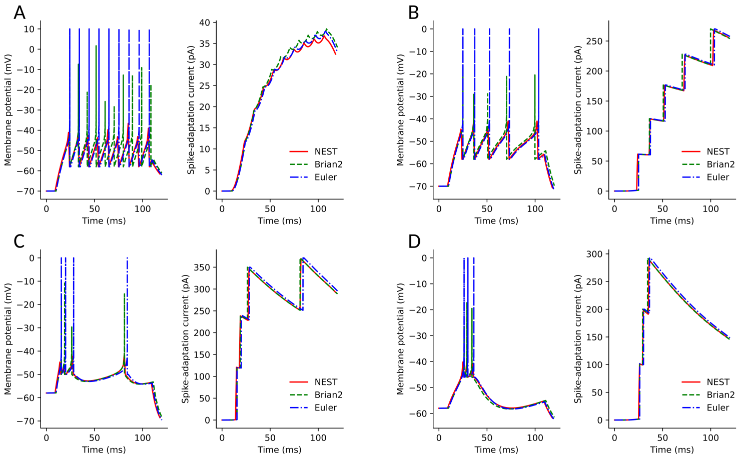

S1 Fig Comparing NEST, Brian 2 and our implementation when using models from [1]. Comparison of three different simulation environments for solving Adaptive Exponential Integrate-and-fire (AdEx) neuron models: NEST (red, solid), Brian 2 (green, dashed), and our own implementation based on the Euler method (blue, dash-dot) when using the models from Figure 4 A-D in [1] which correspond to panel A-D, respectively. The lines represent the membrane potential (left of each panel) and spike-adaptation current (right of each panel) obtained by numerically solving the two equations describing the AdEx. Both NEST and Brian 2 do not draw the full spike, and in case of Brian 2, the slight temporal mismatch arises from the use of its default refractory period.

References

1. Naud R, Marcille N, Clopath C, Gerstner W. Firing patterns in the adaptive exponential integrate-and-fire model. Biol Cybern. 2008 Oct;99:335–347.
