# Supplementary material for: A unified model library maps how neuromodulation reshapes the excitability landscape of neurons across the brain
Source: PLoS Comput Biol. 2025 Dec 1;21(12):e1013765. doi: 10.1371/journal.pcbi.1013765 (PMC12680334; doi:10.1371/journal.pcbi.1013765)
Supplement: S2 Fig — Best 16 cortical pyramidal neuron models in control condition. (PDF) [file pcbi.1013765.s002.pdf]

Supporting information

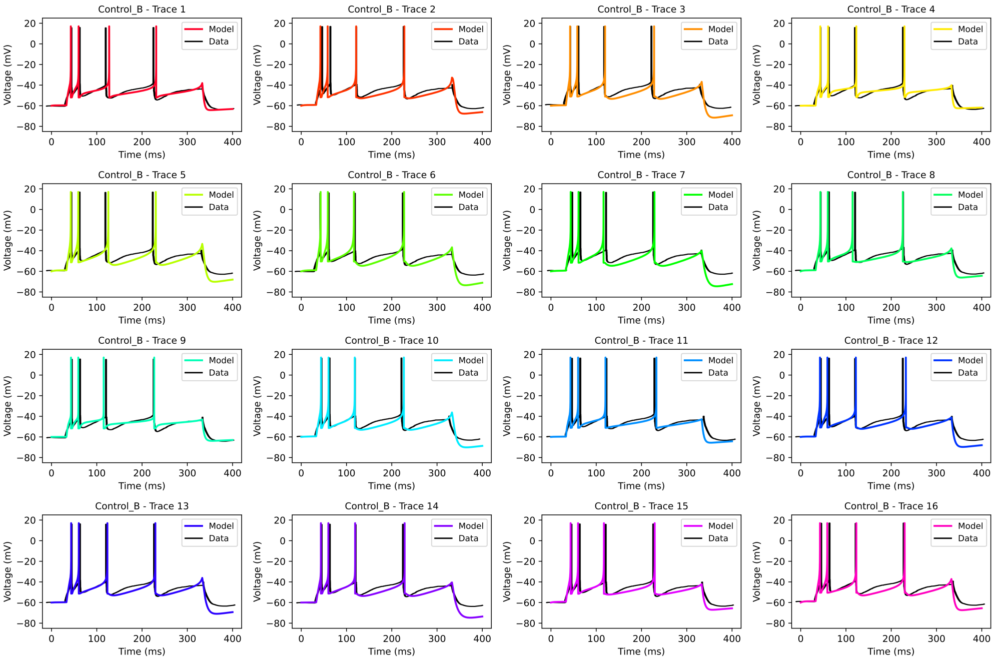

S2 Fig Best 16 **cortical pyramidal neuron models in control condition.** Comparison between the 16 best models (colored lines) and experimental data (back line) extracted from Fig 1 B in [1]. Each subplot shows the membrane potential response for a single model and the experimental voltage recording.

References

1. McCormick DA, Williamson A. Convergence and divergence of neurotransmitter action in human cerebral cortex. Proc Natl Acad Sci U S A. 1989 Oct;86(20):8098–8102.
