# Supplementary material for: A unified model library maps how neuromodulation reshapes the excitability landscape of neurons across the brain
Source: PLoS Comput Biol. 2025 Dec 1;21(12):e1013765. doi: 10.1371/journal.pcbi.1013765 (PMC12680334; doi:10.1371/journal.pcbi.1013765)
Supplement: S3 Fig — Comparison of AdEx model parameters fitted to three electrophysiological traces from the same layer III human cortical neuron. (PDF) [file pcbi.1013765.s003.pdf]

Supporting information

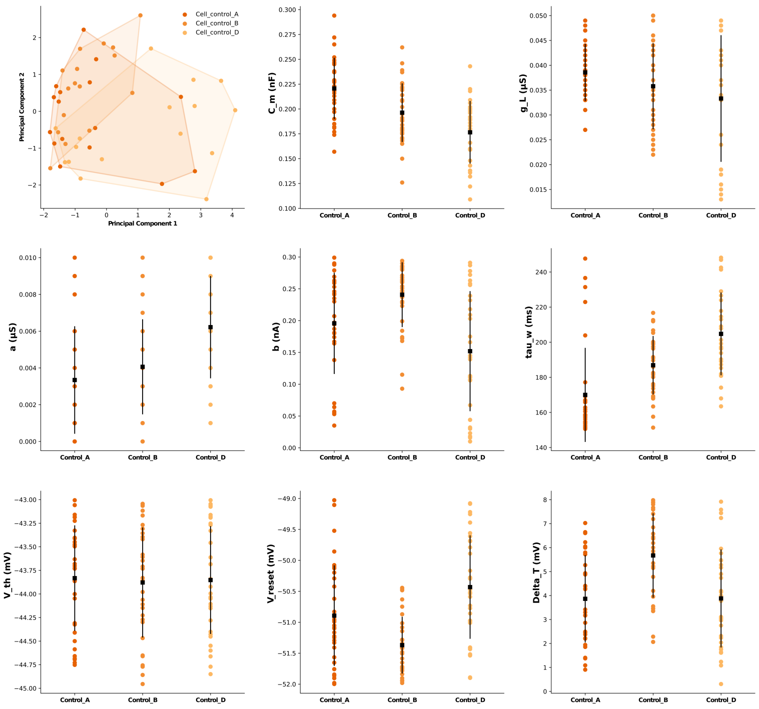

S3 Fig Comparison of AdEx model parameters fitted to three electrophysiological traces from the same layer III human cortical neuron. (Top left) Principal Component Analysis (PCA) of AdEx models independently fitted to three current-clamp traces from [1] (their Fig 1 A, B and D) recorded from the same layer III human cortical neuron using the same current injection protocol. These traces differ by eye, however the parameters of the models they originate are similar. To quantify this similarity, we run the PCA which shows strong overlap between the three clusters (each representing the models obtained from one of the traces), indicating similarity in their underlying parameter structure. Indeed the corresponding silhouette score has a negative value (~ -0.028), indicating indeed poor cluster separation. (Remaining panels) Distribution of selected AdEx parameters (each subplot represents one parameter distribution) where the columns represent the models fitted to one of the three traces, the dots represent single models, while black squares and bars indicate the mean and standard deviation, respectively.

References

1. McCormick DA, Williamson A. Convergence and divergence of neurotransmitter action in human cerebral cortex. Proc Natl Acad Sci U S A. 1989 Oct;86(20):8098–8102.
