# Supplementary material for: A unified model library maps how neuromodulation reshapes the excitability landscape of neurons across the brain
Source: PLoS Comput Biol. 2025 Dec 1;21(12):e1013765. doi: 10.1371/journal.pcbi.1013765 (PMC12680334; doi:10.1371/journal.pcbi.1013765)
Supplement: S4 Fig — The effect of dopaminergic modulation on the subthreshold adaptation parameter. (PDF) [file pcbi.1013765.s004.pdf]

Supporting information

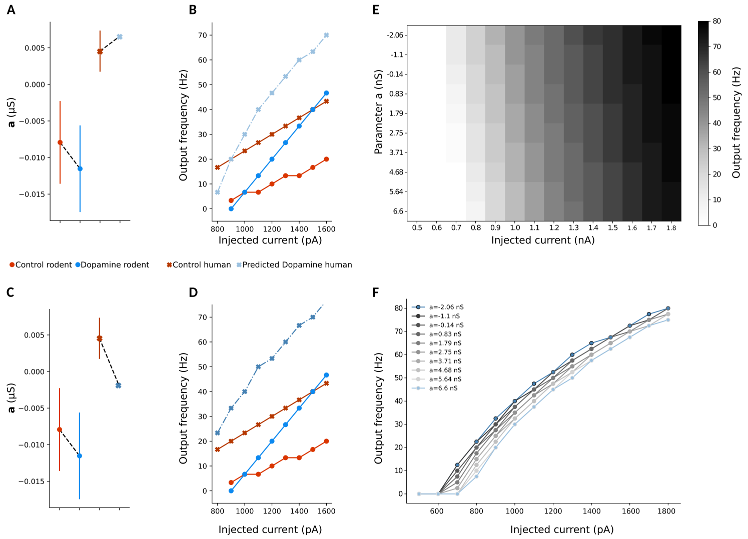

S4 Fig The effect of dopaminergic modulation on the subthreshold adaptation parameter. The parameter a (of the AdEx model) exhibits opposite baseline values in rodents and humans. In rodent control models a is negative (red circle in A and C represents the average) and it further decreases under dopaminergic modulation (blue circle in A and C represents the average). In contrast, human control models (whose average is represented with a red x mark in A and C) show a positive a. This leads to (at least) two possible strategies for predicting the dopaminergic effect on humans: (A) by increasing the absolute value of a and (C) by applying signed decrease. Panels A and C show the resulting changes in a for both strategies, while panels (B) and (D) compare the corresponding output frequency–current curves. Both approaches produce comparable predicted firing dynamics, although the signed decrease (C–D) results in a more pronounced increase in firing frequency. (E) Sensitivity analysis performed using a total of 10 different values of a spanning from the value obtained from the *signed strategy* (-2.06 nS) to that corresponding to the *absolute value strategy* (6.6 nS). Rows correspond to different a while columns to injected current. The shades represent the output firing rate. (F) Output firing frequency as function of injected current for each fixed a value. Interestingly, as the current increases, the influence of a becomes less pronounced. Indeed the variability across curves decreases from about 5 to 2 Hz. This reduction is comparable to the variability typically observed experimentally within a given cell type.
