# Supplementary material for: A unified model library maps how neuromodulation reshapes the excitability landscape of neurons across the brain
Source: PLoS Comput Biol. 2025 Dec 1;21(12):e1013765. doi: 10.1371/journal.pcbi.1013765 (PMC12680334; doi:10.1371/journal.pcbi.1013765)
Supplement: S5 Fig — Summary PCA color-coded by brain region. (PDF) [file pcbi.1013765.s005.pdf]

Supporting information

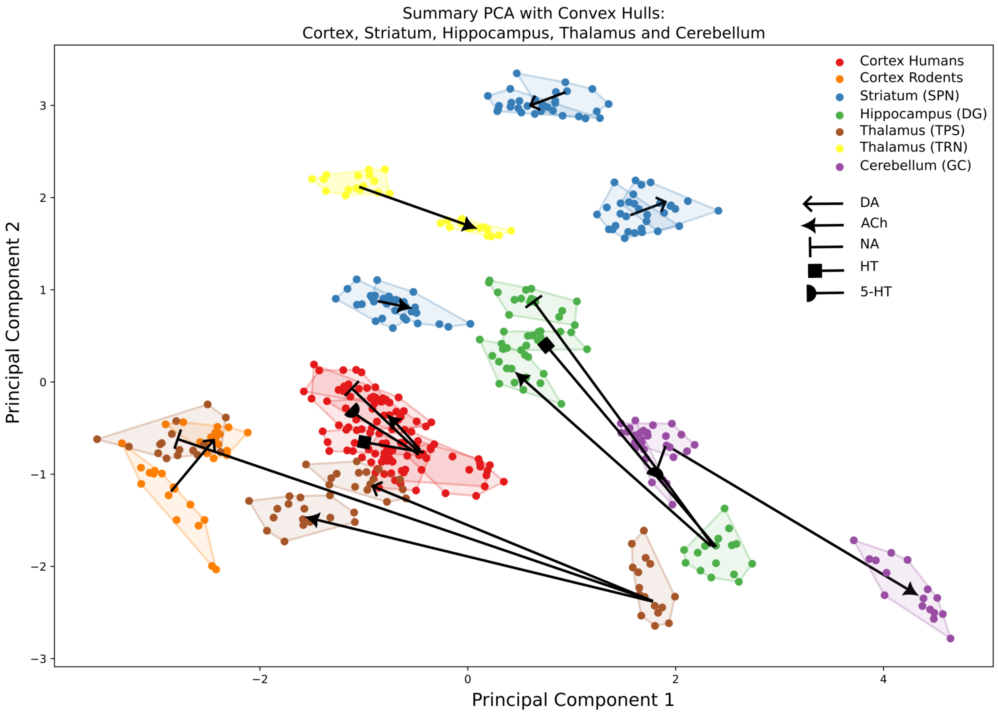

S5 Fig Summary PCA. Each point represents a model, color-coded by brain region. Overall, the data form well-separated clusters. Intrinsic cell properties, such as morphologies, ion channel expression and connectivity, are fundamentally constrained by their region, while neuromodulators, despite reshaping the excitability of the cells, do not override those properties. Arrows connect control and neuromodulated models of the same neuron type; the arrowhead indicates the neuromodulator.
