# Supplementary material for: A unified model library maps how neuromodulation reshapes the excitability landscape of neurons across the brain
Source: PLoS Comput Biol. 2025 Dec 1;21(12):e1013765. doi: 10.1371/journal.pcbi.1013765 (PMC12680334; doi:10.1371/journal.pcbi.1013765)
Supplement: S6 Fig — Summary features-PCA. (PDF) [file pcbi.1013765.s006.pdf]

Supporting information

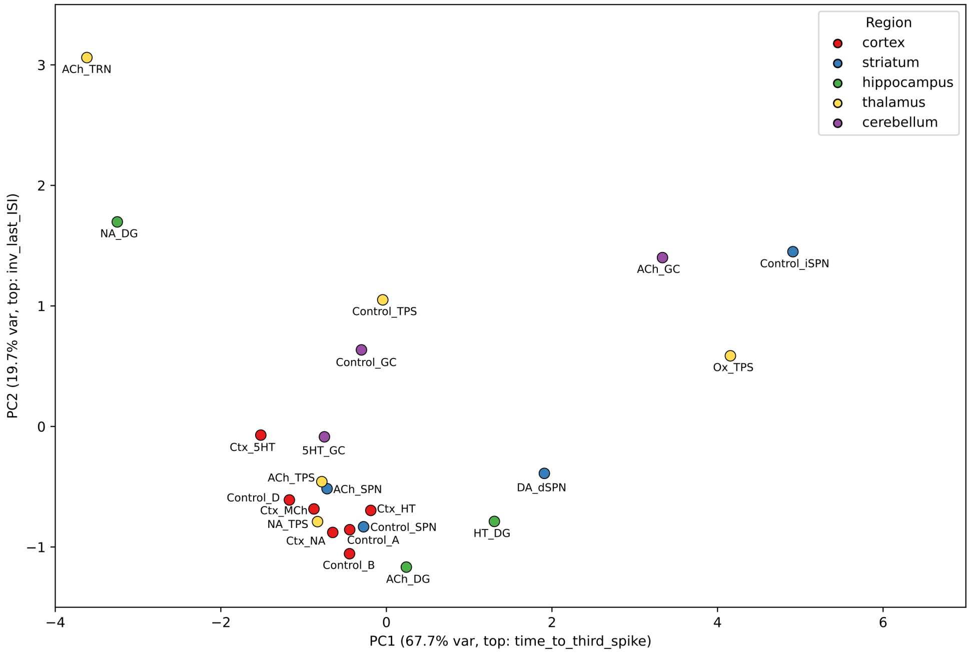

S6 Fig Summary features-PCA. Each point represents a trace color-coded by brain region. The points corresponding to Ctx_Control_A, Ctx_Control_B and Ctx_Control_D are in this case well separated. Ctx_Control_A is much closer to Ctx_NA, and Control_D is much closer to Ctx_MCh than to the other controls. However, when using the AdEx parameters, the three controls overlap appropriately (with a negative Silhouette score, see S3 Fig), reflecting that they correspond to the same cell.

Traces in which at least one feature is `None`, as for example the `time_to_third_spike` in the control direct striatal projection neurons, are excluded from this analysis.
