# Supplementary material for: A unified model library maps how neuromodulation reshapes the excitability landscape of neurons across the brain
Source: PLoS Comput Biol. 2025 Dec 1;21(12):e1013765. doi: 10.1371/journal.pcbi.1013765 (PMC12680334; doi:10.1371/journal.pcbi.1013765)
Supplement: S1 Table — Extended metadata of electrophysiological recordings across brain regions, species, and neuromodulators. (PDF) [file pcbi.1013765.s007.pdf]

S1 Table Extended metadata of electrophysiological recordings across brain regions, species, and neuromodulators.

**Region**	**Species**	**Condition**	**Pub.**	**Temp** (°C)	**Hold. V** (mV)	V_{peak} (mV)	V_{th} (mV)	V_{reset} (mV)	**Hold. I** (pA)	**Protocol** (ms),(pA)
Ctx	Human	Control	[1]	35±1	-60	17	∼ -44	∼ -51	0	[0, 33, 333, 400], [0, 800, 0]
Ctx	Human	Histamine	[1]	35±1	-60	11	∼ -44	∼ -49.5	0	[0, 37, 330, 400], [0, 800, 0]
Ctx	Human	Methacoline	[1]	35 ± 1	-60	15.5	∼ -44	∼-52	0	[0, 37, 335, 400], [0, 800, 0]
Ctx	Human	Norepinephrine	[1]	35 ± 1	-60	11	∼ -45	∼-57	0	[0, 33, 345, 400], [0, 800, 0]
Ctx	Human	Serotonin	[1]	35 ± 1	-60	11	∼ -44	∼-52	0	[0, 33, 330, 400], [0, 800, 0]
Ctx	Rat	Control	[12]	31 − 32	-66	-7.5	∼ -50	∼-51.5	0	[0, 25, 80, 100], [0, 1400, 0]
Ctx	Rat	Dopamine	[12]	31 − 32	-67	-7.5	∼ -46.5	∼-49.5	0	[0, 25, 80, 100], [0, 1400, 0]
St	Mouse	D1_control	[2]	34 − 35	-82	36	∼ -42.5	∼-52	0	[0, 100, 600, 1000], [0, 400, 0]
St	Mouse	D2_control	[2]	34 − 35	-82	42.5	∼ -50	∼-60	0	[0, 100, 600, 1000], [0, 272, 0]
St	Mouse	D1_dopamine	[2]	34 − 35	-82	36	∼ -42.5	∼-53	0	[0, 100, 600, 1000], [0, 400, 0]
St	Mouse	D2_dopamine	[2]	34 − 35	-82	42.5	∼ -50	∼-59	0	[0, 100, 600, 1000], [0, 272, 0]
St	Rat	Control	[3]	34 − 36	-60	10	∼ -52	∼-55	0	[0, 30, 270, 350], [0, 150, 0]
St	Rat	Acetylcholine*	[3]	34 − 36	-60	10	∼ -53	∼-55	0	[0, 32, 260, 350], [0, 150, 0]
Hipp	Mouse	Control	[4]	25	-75	20	∼ -43	∼-49	-300	[0, 37, 537, 600], [-300, 60, -300]
Hipp	Mouse	Control	[4]	25	-75	20	∼ -43	∼-49	-300	[0, 37, 537, 600], [-300, 100, -300]
Hipp	Mouse	Control	[4]	25	-75	20	∼ -43	∼-49	-300	[0, 37, 537, 600], [-300, 140, -300]
Hipp	Mouse	Acetylcholine**	[4]	25	-75	7	∼ -45	∼-53	-200	[0, 37, 537, 600], [-200, 140, -200]
Hipp	Rat	Histamine	[5]	30	-70	9	∼ -45	∼-47	0	[0, 60, 522, 700], [0, 200, 0]
Hipp	Rat	Norepinephrine***	[6]	30	-70	24	∼ -45	∼-55	-40	[0, 17, 93, 150], [-40, 200, -40]
Th	Rat	TPS Control	[7]	32 ± 1	-72	5	∼ -42.5	∼-40	-250	[0, 133, 950, 1650], [-250, 150, -250]
Th	Rat	TPS Orexin-B	[7]	32 ± 1	-60	8	∼ -45	∼-44	-250	[0, 166, 988, 1650], [-250, 150, -250]
Th	Rat	TPS Norepinephrine	[8]	35 ± 1	-60	5	∼ -51	∼-56	-250	[0, 28, 151, 275], [-250, 150, -250]
Th	Rat	TPS Acetylcholine	[8]	35 ± 1	-53	20	∼ -41	∼-48	-250	[0, 28, 151, 275], [-250, 150, -250]
Th	Rat	TRN Control	[9]	36 ± 1	-75	5	∼ -57	∼-64	0	[0, 10, 43, 100], [0, 250, 0]
Th	Rat	TRN Acetylcholine	[9]	36 ± 1	-75	5	∼ -52	∼-55	0	[0, 11.5, 47, 100], [0, 250, 0]
Cb	Rat	Control	[10]	31 − 33	-70	5	∼ -32	∼-39	0	[0, 54, 191, 300], [0, 16, 0]
Cb	Rat	Norepinephrine	[10]	31 − 33	-70	5	∼ -38	∼-41	0	[0, 54, 191, 300], [0, 16, 0]
Cb	Rat	Acetylcholine*	[11]	21 − 23	-71	30	∼ -19	∼-20	0	[0, 116, 458, 600], [0, 100, 0]

* Muscarine, a selective agonist of Acetylcholine was bath applied.
** Oxo-M, a muscarinic agonist was bath applied.
*** Isoproterenol, a β-adrenergic agonist was bath applied.

References

1. McCormick DA, Williamson A. Convergence and divergence of neurotransmitter action in human cerebral cortex. Proc Natl Acad Sci U S A. 1989 Oct;86(20):8098–8102.

2. Planert H, Berger TK, Silberberg G. Membrane properties of striatal direct and indirect pathway neurons in mouse and rat slices and their modulation by dopamine. PLoS One. 2013;8(3):e57054.

3. Perez-Rosello T, Figueroa A, Salgado H, Vilchis C, Tecuapetla F, Guzman JN, Galarraga E, Bargas J. Cholinergic control of firing pattern and neurotransmission in rat neostriatal projection neurons: role of $Ca_V2.1$ and $Ca_V2.2$ Ca^{2+} channels. J Neurophysiol. 2005;93(5):2507–2519.

4. Carver CM, Shapiro MS. Gq-coupled muscarinic receptor enhancement of KCNQ2/3 channels and activation of TRPC channels in multimodal control of excitability in dentate gyrus granule cells. J Neurosci. 2019;39(9):1566–1587.

5. Greene RW, Haas HL. Effects of histamine on dentate granule cells in vitro. Neuroscience. 1990;34(2):299–303.

6. Haas HL, Rose GM. Noradrenaline blocks potassium conductance in rat dentate granule cells in vitro. Neurosci Lett. 1987;78(2):171–174.

7. Govindaiah G, Cox CL. Modulation of thalamic neuron excitability by orexins. Neuropharmacology. 2006;51(3):414–425.

8. McCormick DA. Cholinergic and noradrenergic modulation of thalamocortical processing. Trends Neurosci. 1989;12(6):215–221.

9. McCormick DA, Prince DA. Acetylcholine induces burst firing in thalamic reticular neurones by activating a potassium conductance. Nature. 1986;319(6052):402–405.

10. Fleming E, Hull C. Serotonin regulates dynamics of cerebellar granule cell activity by modulating tonic inhibition. J Neurophysiol. 2019;121(1):105–114.

11. Watkins CS, Mathie A. A non-inactivating K+ current sensitive to muscarinic receptor activation in rat cultured cerebellar granule neurons. J Physiol. 1996;491(2):401–412.

12. Onn SP, Wang XB, Lin M, Grace AA. Dopamine D1 and D4 receptor subtypes differentially modulate recurrent excitatory synapses in prefrontal cortical pyramidal neurons. Neuropsychopharmacology. 2006;31(2):318–338.
