# Supplementary material for: A unified model library maps how neuromodulation reshapes the excitability landscape of neurons across the brain
Source: PLoS Comput Biol. 2025 Dec 1;21(12):e1013765. doi: 10.1371/journal.pcbi.1013765 (PMC12680334; doi:10.1371/journal.pcbi.1013765)

Supporting information

S1 Appendix. Comparing AdEx parameters. In this appendix, we provide a comparative analysis of AdEx parameters across control and neuromodulated conditions for seven neuron types: human and rodent cortical pyramidal neurons, striatal direct and indirect projection neurons, dentate gyrus neurons, thalamocortical projecting neurons, thalamic reticular neurons, and cerebellar Granule neurons. For each neuron type and condition, we display the distributions of the AdEx parameters corresponding to the top 16 models, alongside their means and standard deviations. Last panel represents the parameters of human and rodent cortical models, their averages, standard deviations, and the DA-induced shifts (with respect to the control).

Human Cortical Pyramidal Neurons

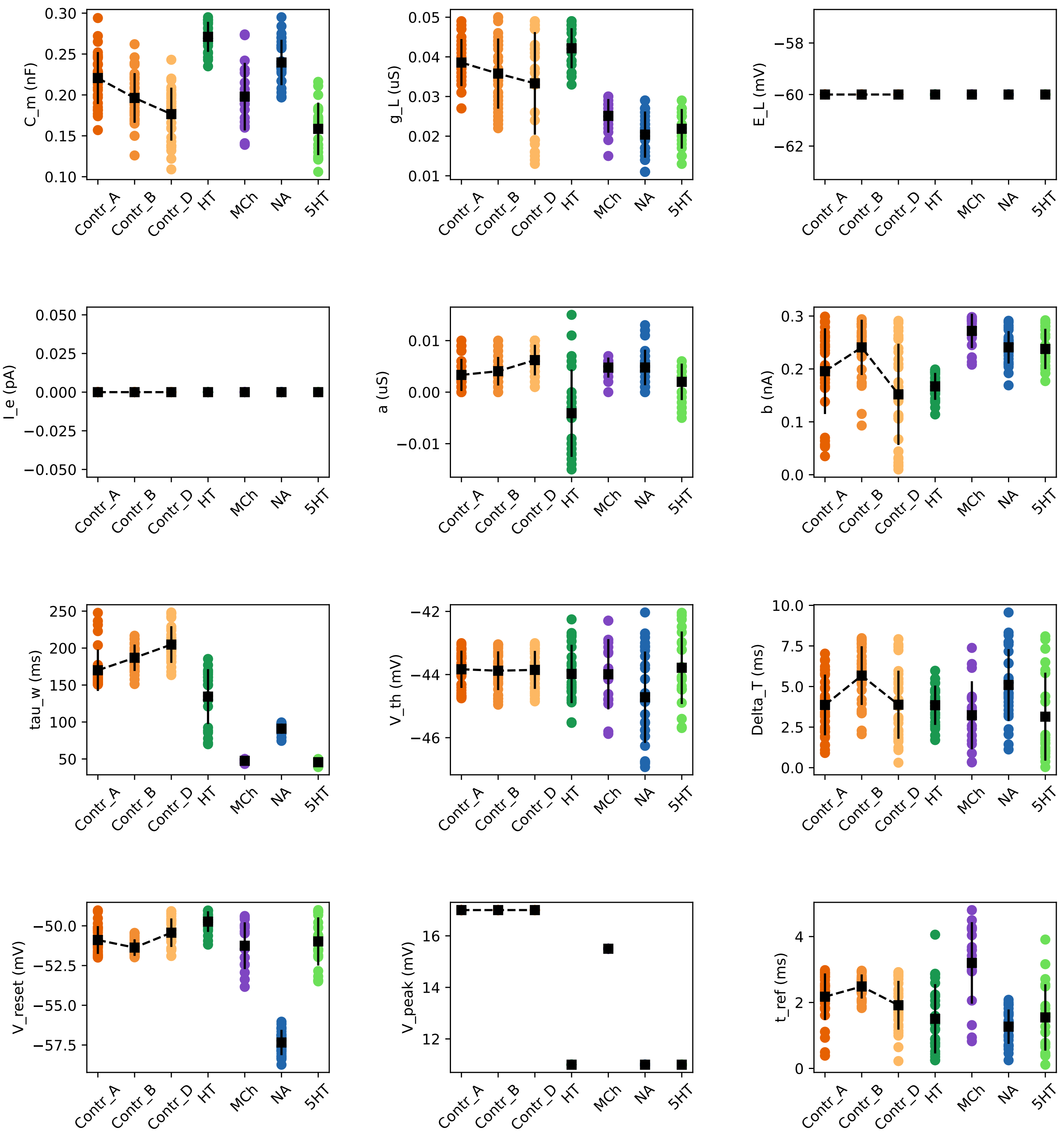

Rat Cortical Neurons

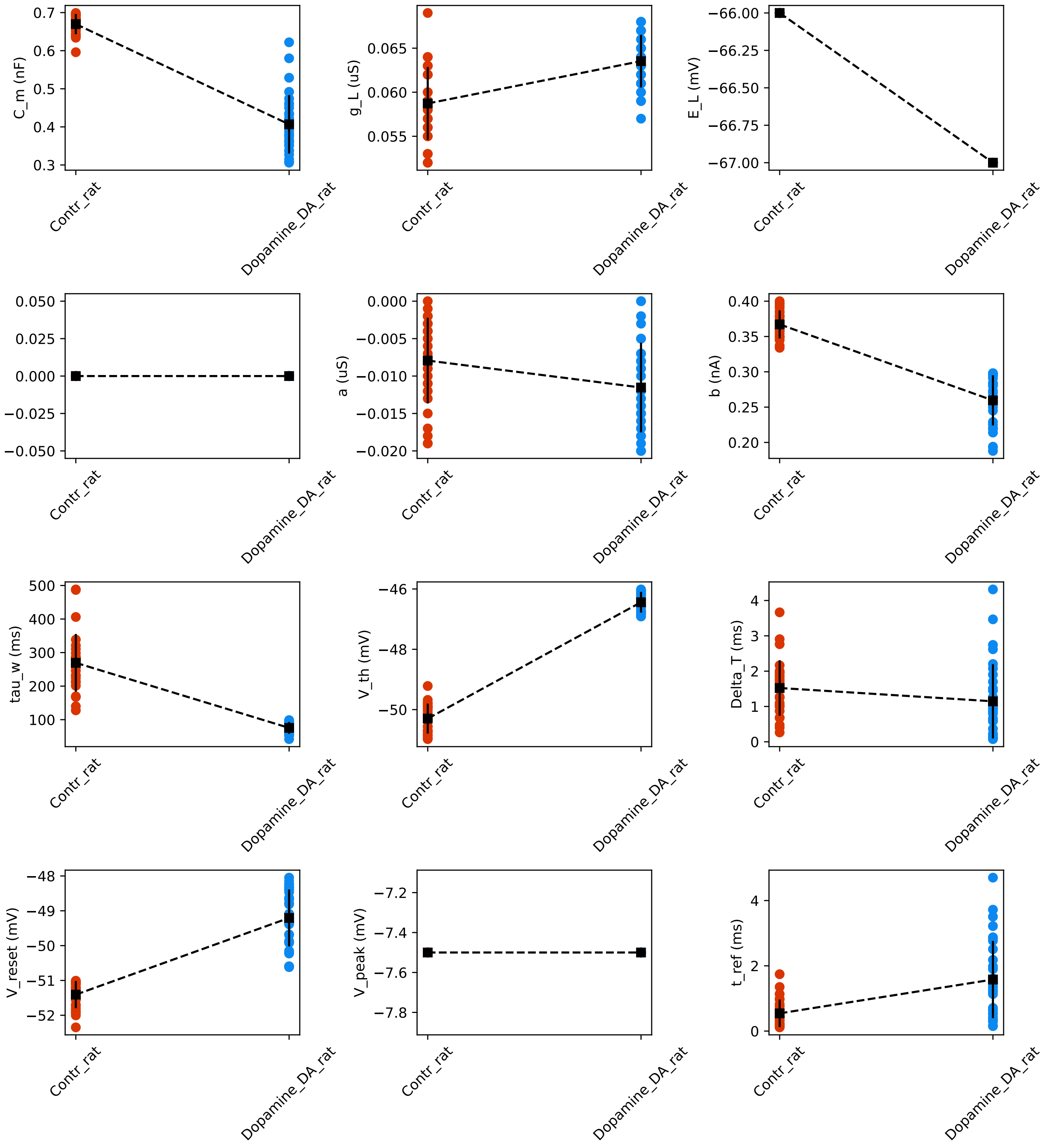

Striatal Projection Neurons

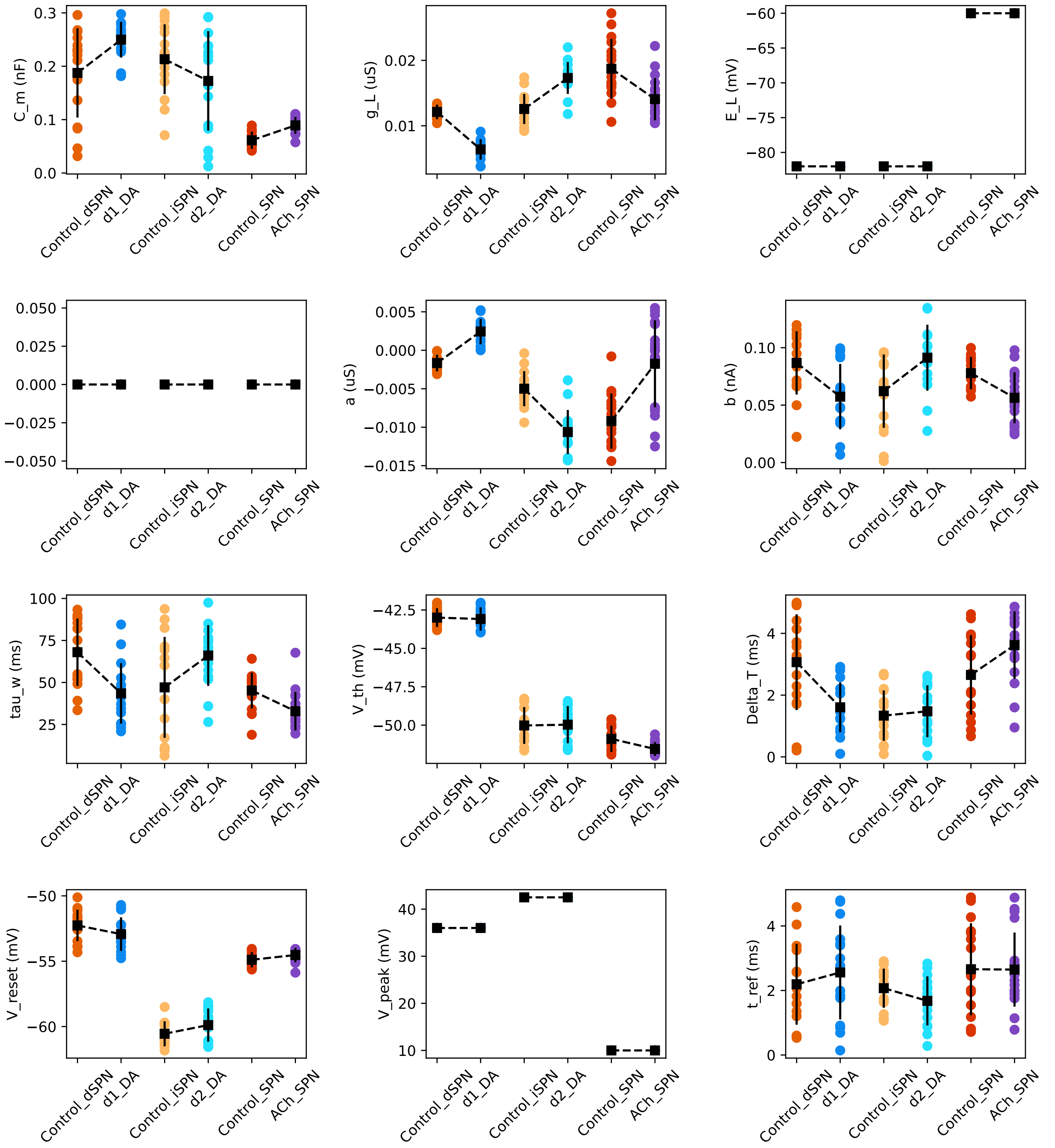

Dentate Gyrus Neurons

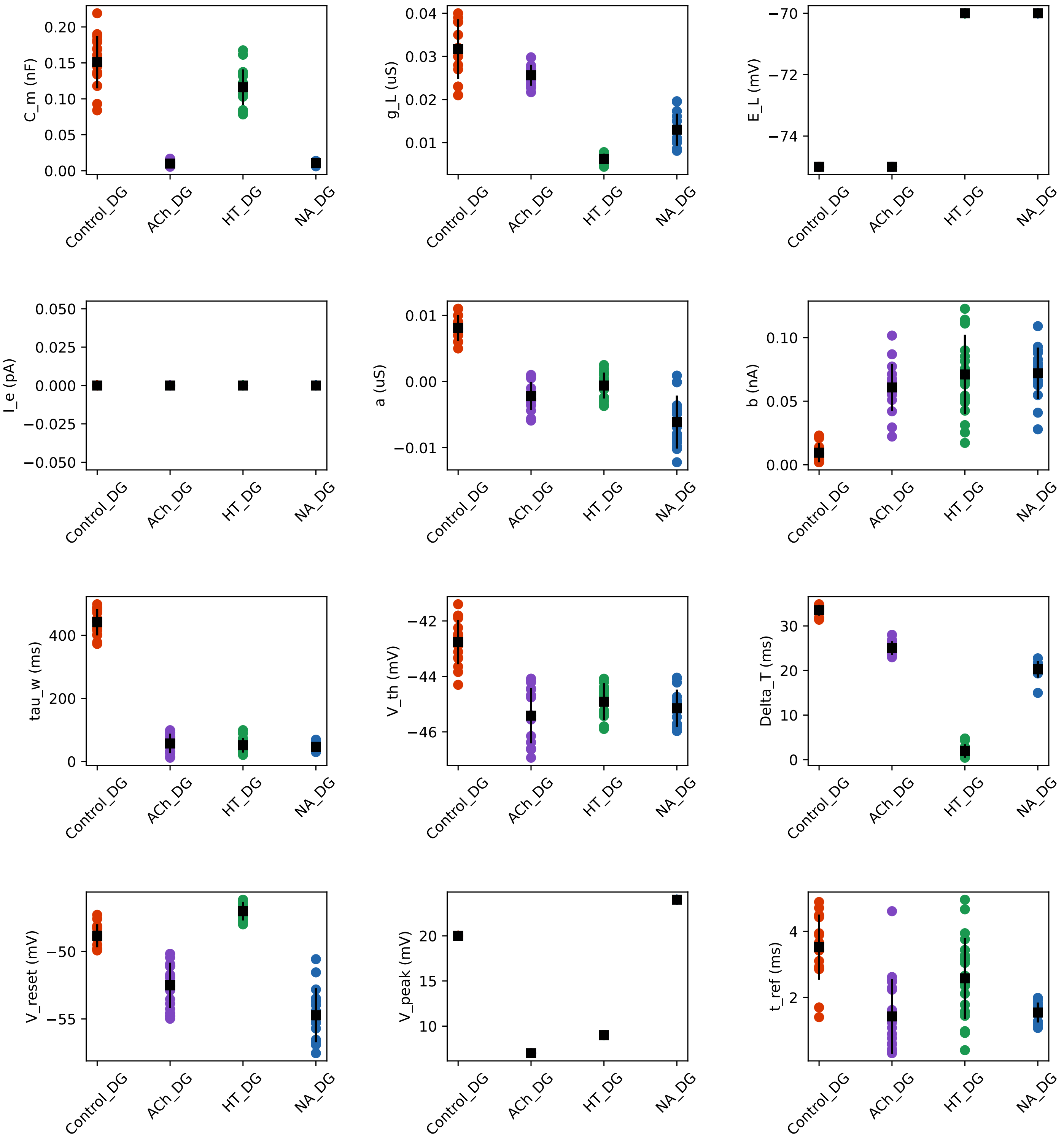

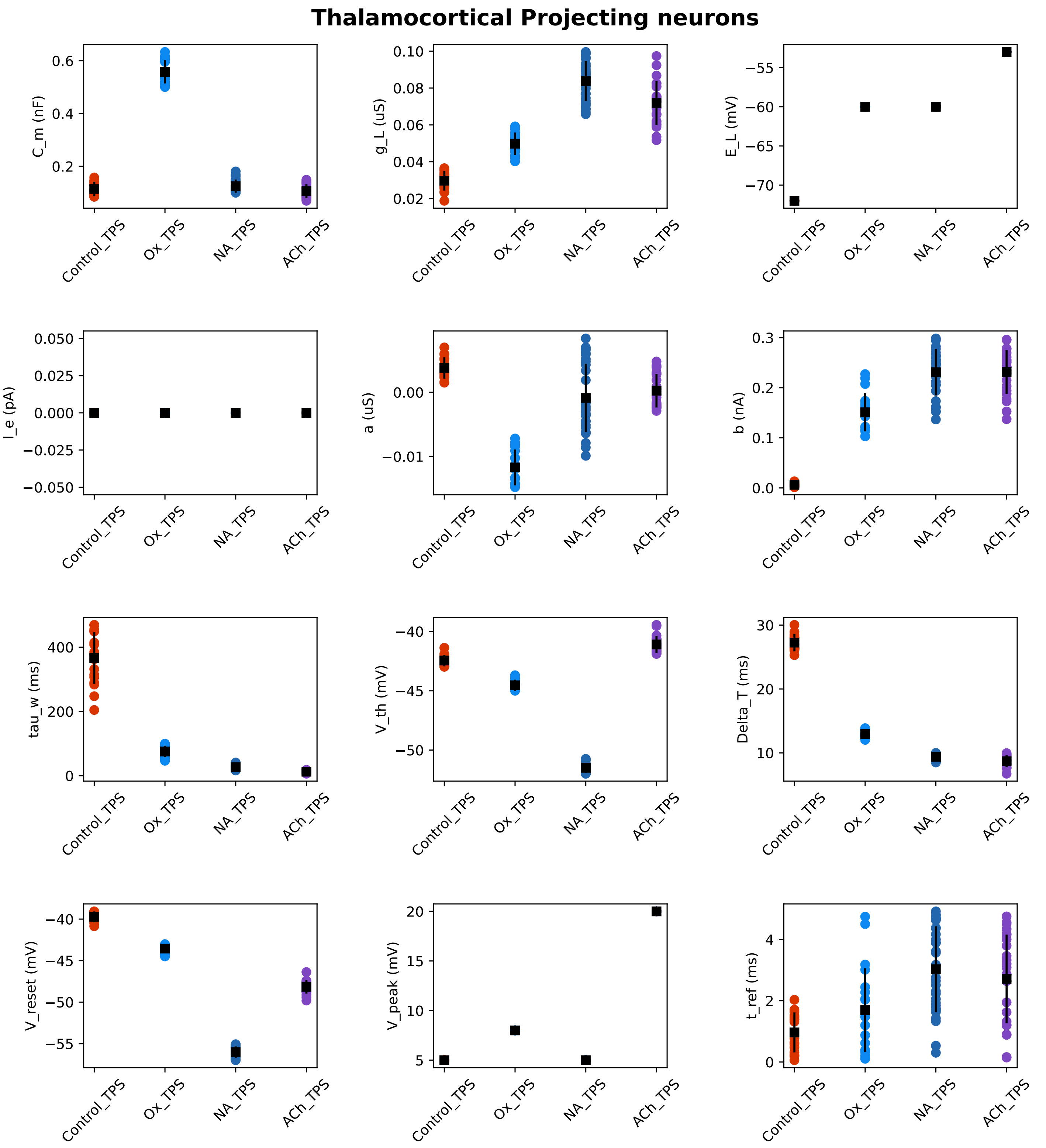
Thalamocortical Projecting neurons
C_m (nF)
g_L (uS)
E_L (mV)
I_e (pA)
a (uS)
b (nA)
tau_w (ms)
V_th (mV)
Delta_T (ms)
V_reset (mV)
V_peak (mV)
t_ref (ms)
Control_TPS
Ox_TPS
NA_TPS
ACh_TPS

Thalamic reticular neurons

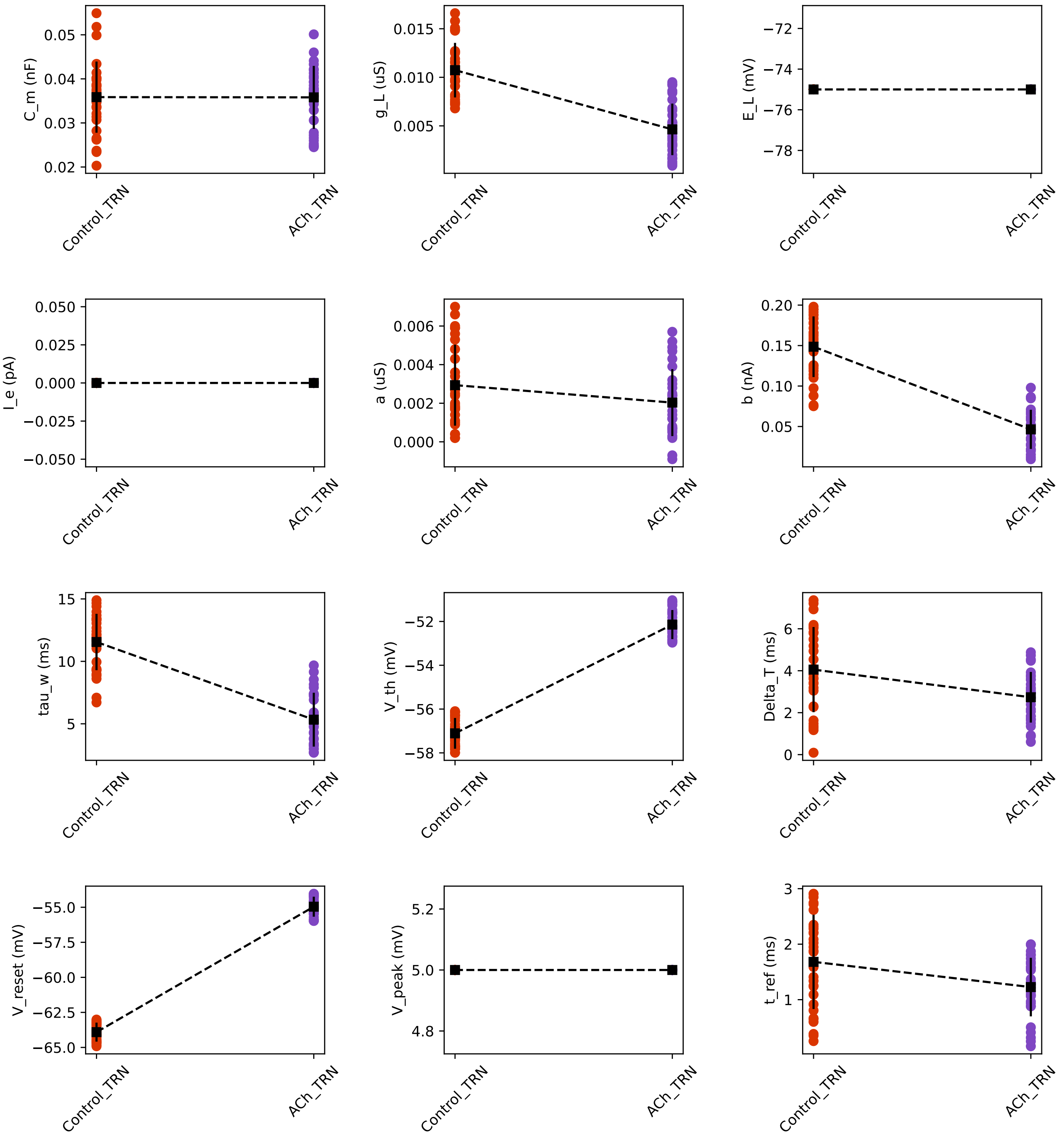

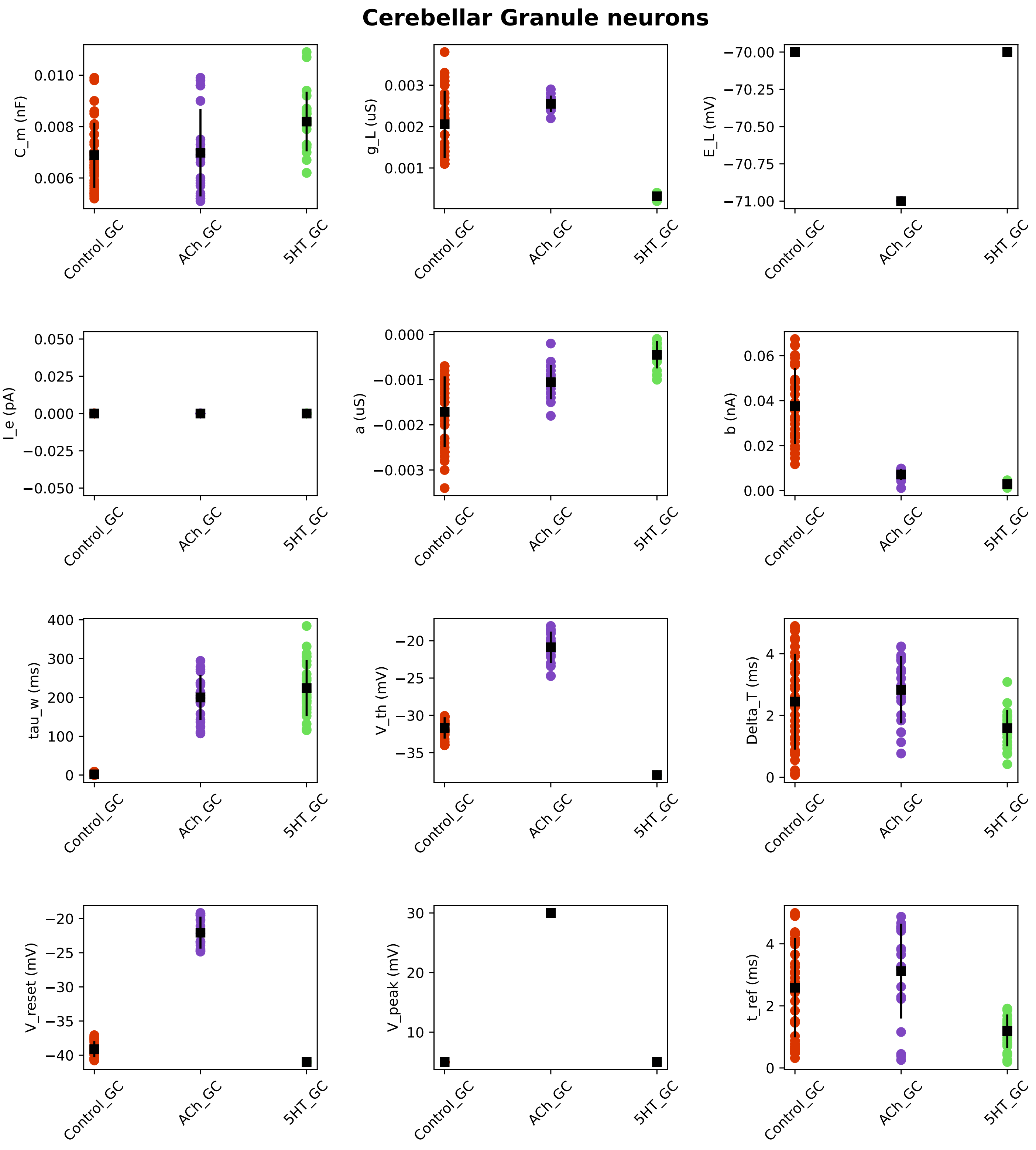
Cerebellar Granule neurons
C_m (nF)
g_L (uS)
E_L (mV)
I_e (pA)
a (uS)
b (nA)
tau_w (ms)
V_th (mV)
Delta_T (ms)
V_reset (mV)
V_peak (mV)
t_ref (ms)
Control_GC
ACh_GC
5HT_GC

Dopamine prediction

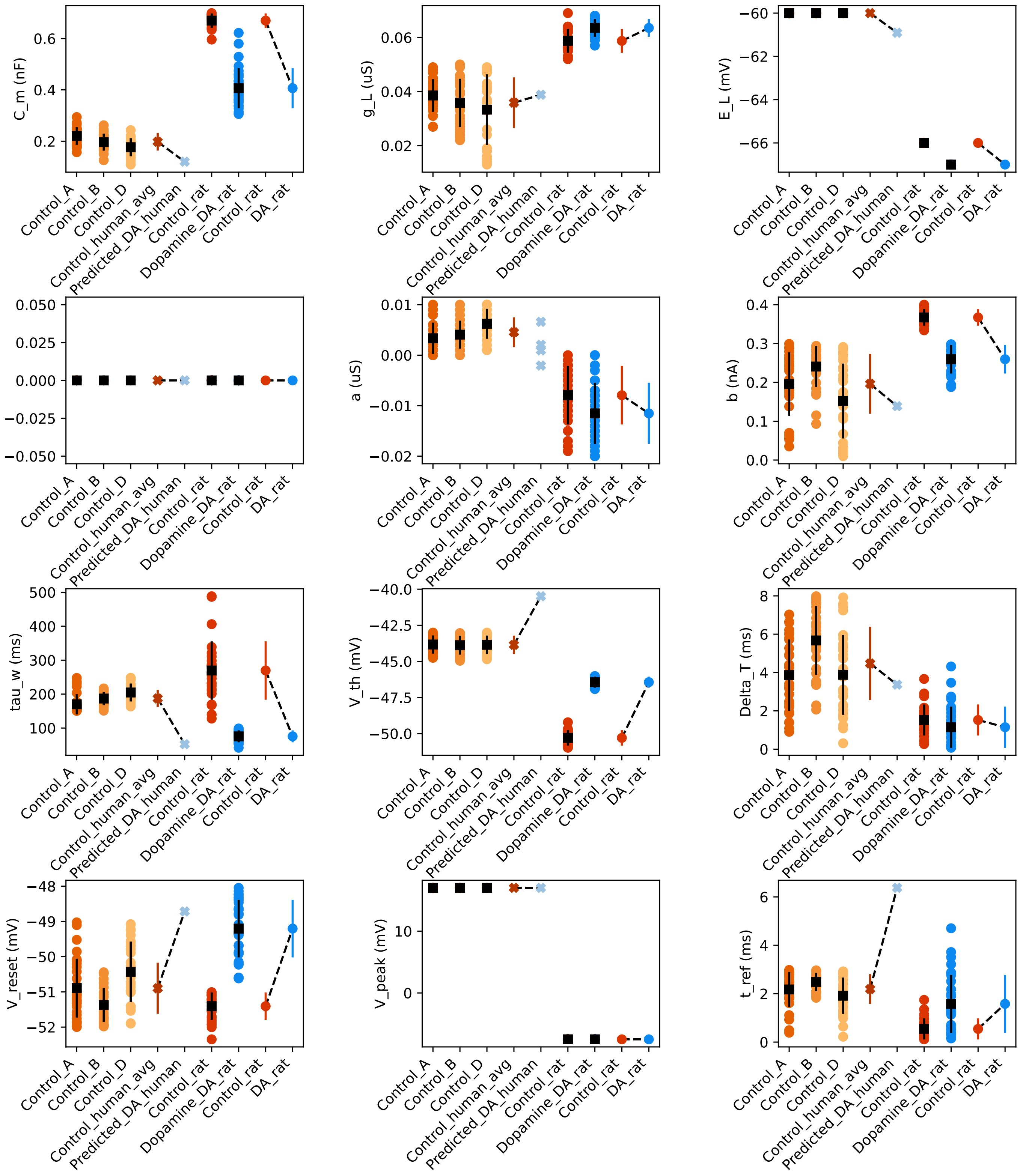

Supplement: S1 Appendix — Comparing AdEx parameters. (PDF) [file pcbi.1013765.s008.pdf]
