# Supplementary material for: A unified model library maps how neuromodulation reshapes the excitability landscape of neurons across the brain
Source: PLoS Comput Biol. 2025 Dec 1;21(12):e1013765. doi: 10.1371/journal.pcbi.1013765 (PMC12680334; doi:10.1371/journal.pcbi.1013765)
Supplement: Attachment — Submitted filename: remarks-to-authors.pdf [file pcbi.1013765.s009.pdf]

Thank you for sending me this interesting article to review!

Neuromodulators regulate neuronal excitability and synaptic plasticity in every corner of the central nervous system, suggesting that they play at least some type of role in essentially every aspect of neural computation. Unfortunately, this broad involvement coupled with a dearth of relevant publically available data seems to have meant that neuromodulation is sometimes incorporated into computational models in a fairly unconstrained and ad hoc manner. By presenting a concise, model-based summary of the effects of multiple neuromodulators in a range of cell types, this work represents an important step towards a clearer understanding of the role of neuromodulation in neural computation.

The main contributions of this work are as follows: First, the authors describe a practical and robust technique for fitting models of neuromodulation to electrophysiological voltage traces from published papers (the only reliably publically available source of relevant data). Second, the authors provide an encyclopaedic overview of the effects of five different neuromodulators in seven different cell types. Third, the authors provide software and model parameters as a resource for other modellers to use in their own simulations.

Overall, this work promises to be useful both as a reference/model bank and as a guide for fitting models to realistic amounts of data. My only major concern is that I was not able to access the GitHub repository (is it still private?) or find another way to view the code, data, and fitted models that collectively form one of the main contributions of this work. While the paper itself is generally very nice, I'd like to offer a few minor comments and suggestions for the authors to consider.

Presentation

The paper is written in a clear and didactic style and the figures are nicely put together. However, the manuscript could be made more concise by reducing repetition. In particular, the first few paragraphs of the results felt like a medium-length summary of the methods on my first read through. I'm also not sure that the explanation of the ionic basis of neural excitability in the second paragraph of the introduction is necessary.

If the authors can reduce the overall length of the text, they could consider using some of this space to add an introductory figure. This figure could function almost like a graphical abstract, giving a high level overview of the systematic review and model fitting process as well as illustrating the components of the AdEx model and how they contribute to different firing patterns. Part of this figure could include a simple visual guide on how to interpret the phase plots. While this has of course been done elsewhere, the phase plots are such an important part of the figures in this paper that including something along the lines of Figs. 6.3 or 6.4 from the Gerstner *Neuronal Dynamics* textbook might help make the manuscript more self-contained and accessible.

Finally, the title of the paper seems tautological and not very reflective of the descriptive nature of the work. It would be a shame for someone looking for a bank of neuromodulation models to miss this work!

Technical/scientific

- L66-89: "Data collection and curation" glosses over a lot of work. For a systematic review, I would normally expect to see a full list of the search queries used and a flow chart detailing the number of papers considered and their reasons for inclusion/exclusion. Perhaps this could be included in supplementary material?
- L109-111: Was any debouncing used to ensure that detected ISIs were not shorter than eg a few ms?
- L114-115: Number of spikes over the entire stimulus duration should be interpreted differently depending on the length of the stimulus (eg apparent firing rates for a 100ms vs. 1000ms stimulus might be very different for a strongly adapting neuron). Maybe the authors could report any significant variation in stimulus length across studies?
- L171-177: What time step size was used for Euler integration?
- L219-227: Membrane potential was weighted more heavily than other features in the loss function. Were the rest of the features equally-weighted? Could be more explicit.

- L451: I'm quite surprised that Gi-coupled 5-HT1A is excitatory, especially since this is the receptor responsible for the inhibitory effects of serotonin on serotonin neurons. If excitation is the exception rather than the rule as I suspect, this would be good to note.
- Neural excitability can be strongly temperature dependent. It would be helpful for the authors to include the recording temperature in table 1.
- It seems like the level of input current used to calculate the voltage nullclines isn't reported. I assume zero, but this would mean that the phase plots correspond to a state where the neuron isn't spiking (consistent with the presence of a stable fixed point at resting membrane potential), unlike the sample traces. I found this discrepancy a bit confusing.
- I couldn't find the exact PC loadings anywhere.
- How were the centroid models obtained? Are they the sampled model closest to the center of the convex hulls shown in the PC figures? Or are they averages of the top 16 models?
- L753-756: The general approach of using prior information to constrain the parameter search space and reporting a distribution of models rather than a single best-fitting option feels a bit Bayesian in spirit. Perhaps the authors could comment on this?

Typos

- L120-126: equations seem to be missing parentheses for the denominator.
- "(see <blank>)" in various places. L247, L744
