# Supplementary material for: A unified model library maps how neuromodulation reshapes the excitability landscape of neurons across the brain
Source: PLoS Comput Biol. 2025 Dec 1;21(12):e1013765. doi: 10.1371/journal.pcbi.1013765 (PMC12680334; doi:10.1371/journal.pcbi.1013765)
Supplement: Attachment — Submitted filename: PCOMPBIOL-D-25-01115_response_to_reviewers.pdf [file pcbi.1013765.s011.pdf]

PCOMPBIOL-D-25-01115

Answers to Reviewers' comments

We would like to thank the reviewers for their constructive and insightful comments, which helped us improve the manuscript.

We refer to the manuscript (without track changes) for the line numbers.

Reviewer 1:

- My only major concern is that I was not able to access the GitHub repository (is it still private?) or find another way to view the code, data, and fitted models that collectively form one of the main contributions of this work.
  - Yes, the GitHub repository was still private, we apologize for this. We have now made it publicly available.
- The paper is written in a clear and didactic style and the figures are nicely put together. However, the manuscript could be made more concise by reducing repetition. In particular, the first few paragraphs of the results felt like a medium-length summary of the methods on my first read through. I'm also not sure that the explanation of the ionic basis of neural excitability in the second paragraph of the introduction is necessary. If the authors can reduce the overall length of the text, they could consider using some of this space to add an introductory figure. This figure could function almost like a graphical abstract, giving a high level overview of the systematic review and model fitting process as well as illustrating the components of the AdEx model and how they contribute to different firing patterns. Part of this figure could include a simple visual guide on how to interpret the phase plots. While this has of course been done elsewhere, the phase plots are such an important part of the figures in this paper that including something along the lines of Figs. 6.3 or 6.4 from the Gerstner Neuronal Dynamics textbook might help make the manuscript more self-contained and accessible.
  - We reduced the text, removing all the more didactic material. We removed the ionic bases of neuromodulation from the introduction, and we shortened the beginning of the results section.
  - We thank the reviewer for suggesting to add an introductory figure / graphical abstract. Although we did not include a separate new figure, we have substantially improved Figure 1 to address this helpful suggestion. Specifically, we split the original panel D into two separate panels (now panels E and F), where we show both the phase plane direction field and the streamlines. In addition, we added a new analysis, the excitability landscape, which is now presented as panel D. For the subsequent figures in the manuscript, panels D, E, and F are condensed into a single panel (to maintain readability without adding distinct panels to already dense figures), while the full details are provided in the corresponding Jupyter notebooks available on GitHub.
  - We now included, in all main figures, an excitability landscape panel, where we represent the excitability class of each cell type and how it changes from

the control to neuromodulated conditions, and we also present the corresponding phase space panels as insets. As suggested by the Reviewer, we introduced streamlines in all Jupyter Notebooks, where we draw each phase plot separately, to better appreciate the changes induced by each neuromodulator.

- Finally, the title of the paper seems tautological and not very reflective of the descriptive nature of the work. It would be a shame for someone looking for a bank of neuromodulation models to miss this work!
  - We thank the reviewer for this suggestion, we agree that the title was not sufficiently descriptive.
    We have now included different options that better reflect the content of the work and will make it easier for researchers searching for neuromodulation models to find our paper. Which alternative do you prefer?
- L66-89: "Data collection and curation" glosses over a lot of work. For a systematic review, I would normally expect to see a full list of the search queries used and a flow chart detailing the number of papers considered and their reasons for inclusion/exclusion. Perhaps this could be included in supplementary material?
  - We clarified this point in the text (at lines 71-83). We used a single rich PubMed query to avoid biases due to the adoption of different queries. The only changing term was the <neuromodulator>. The only inclusion/exclusion criterion was the presence of at least one electrophysiology trace for the control and modulated conditions, to allow the model fitting.
- L109-111: Was any debouncing used to ensure that detected ISIs were not shorter than eg a few ms?
  - We did not need to apply any debouncing technique because we didn't have access to any raw recordings, and the data we analysed was already "pre-processed". They were quite clean since they had already been used in previous publications to showcase the behaviour of specific neurons and the effects of neuromodulators. However, since the number of available traces was unfortunately limited, we manually verified spike detection by plotting the detected spike times as markers over the voltage traces, and no unrealistic short ISIs were present.
- L114-115: Number of spikes over the entire stimulus duration should be interpreted differently depending on the length of the stimulus (eg apparent firing rates for a 100ms vs. 1000ms stimulus might be very different for a strongly adapting neuron). Maybe the authors could report any significant variation in stimulus length across studies?
  - Excellent observation. It would have been ideal to have traces with at least 1s stimulation. In our dataset, the stimulus duration ranges from approximately 30 ms to 820 ms. The exact stimulation protocol and the holding voltage value for each trace are reported in the metadata .csv file available on GitHub at:
    (https://github.com/Computational-NeuroPSI/Neuromodulation/blob/main/data/data_traces/data_collection_curation.csv).
    We have now included this information as a table in the Supplementary (Sup Table 1).
    Despite this quite large span, the two traces which express strong spike-frequency adaptation, namely Dentate granule cells, Control and

Thalamocortical Projecting Neurons, Control, were recorded with relatively long stimuli (500 ms and 820 ms, respectively) with the last spike arriving within the half duration of the current injection. The traces recorded using shorter stimuli are fairly regular, so in both cases the estimate of the frequency is reliable.

- L171-177: What time step size was used for Euler integration?
    - We used a time step of 0.01 ms for Euler integration. We could have selected a much coarse resolution, even 0.1 ms, as the result was already qualitatively accurate enough. However, since there were no significant slowdowns we adopted a finer one. Nonetheless, the value of the time step size (dt) can be easily set by the user.
- L219-227: Membrane potential was weighted more heavily than other features in the loss function. Were the rest of the features equally-weighted? Could be more explicit.
    - Only in the two "strong adaptation cases" the error in relation to the value of the membrane potential at the end of the stimulus was weighted more than those corresponding to other features. In all other cases, all the features were weighted equally. However, we have now improved the code, and the user can change the weights depending on their own needs.
      We have clarified this at lines 237-243.
- L451: I'm quite surprised that Gi-coupled 5-HT1A is excitatory, especially since this is the receptor responsible for the inhibitory effects of serotonin on serotonin neurons. If excitation is the exception rather than the rule as I suspect, this would be good to note.
    - Thanks for pointing this out. Indeed, the receptor is 5-HT_{2A}. The inclusion of the 1A was an oversee due to the inclusion of both receptors in the original article exploring the effects of Serotonin. We have now fixed the text (and also removed the reference to the receptor 5-HT_{1A}).
- Neural excitability can be strongly temperature dependent. It would be helpful for the authors to include the recording temperature in table 1.
    - We agree with the reviewer and we added the temperature at recording time as reported in the referenced papers in Table 1, Supplementary Table 1 (an extended version of Table 1), and metadata file.
- It seems like the level of input current used to calculate the voltage nullclines isn't reported. I assume zero, but this would mean that the phase plots correspond to a state where the neuron isn't spiking (consistent with the presence of a stable fixed point at resting membrane potential), unlike the sample traces. I found this discrepancy a bit confusing.
    - The input current used to calculate the nullclines is not zero; it varies across cells, and corresponds to the values reported in the referenced articles. These details are provided in the Jupyter notebooks, and we chose not to include them in the main text to keep it concise. As a result, the phase plots do not correspond to a pure resting state. Instead, they show all possible states, with the resting state appearing as one attractor fixed point, and they capture the full range of voltage dynamics, including the sample traces. We wonder whether the reviewer's comment reflects a misunderstanding of our explanation, or whether we misunderstood the reviewer's comment.
- I couldn't find the exact PC loadings anywhere.

    - This information is indeed not included in the main text for brevity, but it is present in the jupyter notebooks. Specifically we have a jupyter notebook for each brain region and they provide the full loading matrices allowing for in-depth examination.
- How were the centroid models obtained? Are they the sampled model closest to the center of the convex hulls shown in the PC figures? Or are they averages of the top 16 models?
    - The centroid models, shown as full colored dots in the PCA plots, represent the model closest to the center of the convex hulls with the exception of Figure 8 where they are generated from the averages. Specifically for the "human control" the center represents the model derived from the averages between the parameters of the models corresponding to the 3 control traces (3 times 16 best models). While for "rodent control" and "rodent dopamine" they represent the model obtained by averaging the parameters of the best 16 models.
- L753-756: The general approach of using prior information to constrain the parameter search space and reporting a distribution of models rather than a single best-fitting option feels a bit Bayesian in spirit. Perhaps the authors could comment on this?
    - This is an interesting point, we did not consider. Our optimisation starts with biologically informed parameter ranges (a de-facto prior), then samples that space quasi-uniformly (Sobol) and retains the parameter sets whose simulated features fall within a tolerance of the experimental data. The resulting ensemble behaves much like an approximate posterior: it encodes parameter uncertainty and lets us propagate that uncertainty to any derived quantity (f-I curves, phase-plane structure, etc.). Although we stop short of a fully Bayesian treatment (there is no likelihood function or MCMC), our procedure shares two Bayesian aspects:
        - Prior knowledge constrains exploration. Bounds taken from the literature keep the search focused on physiologically plausible regions, just as an explicit prior would.
        - Inference yields a distribution, not a point estimate. Rather than collapsing to a single 'best' model, we report the entire cloud of admissible solutions, analogous to drawing samples from a posterior.

      A stricter Bayesian formalism, our workflow could be reframed as an Approximate Bayesian Computation (ABC) scheme:
      - the Sobol sequence becomes the ABC proposal step;
      - the feature-space error is the ABC distance metric;
      - the acceptance threshold controls posterior width.

      Moving to a full ABC-SMC or MCMC implementation would require: defining an explicit likelihood or kernel, and updating the sampling strategy. We chose the current approach for computational efficiency and ease of interpretation, but the code base is modular and could accommodate a more traditional Bayesian loop if future work calls for it.
- Typos:
- L120-126: equations seem to be missing parentheses for the denominator.
    - Thank you for noticing this. It is now corrected.
- "(see <blank>)" in various places. L247, L744

- Thank you for noticing this as well. They are all references to the S1 Appendix (L247, L506, L696 and L744), but there was a compiling error. It is now fixed.

Reviewer 2:

Comments in mail

- The topic is interesting and the manuscript has potential, though it could still be improved regarding both analysis and presentation. The manuscript states that it aims to understanding neuromodulators, while at the moment it only brings the complexity of neuromodulation to another level, the parameter space, without sorting neuromodulatory effects. The approach to fit models to study the effect on the dynamics across cell types and species, the use of a set of models instead of a single one, and the detail of the model is appropriate for the research question. However, in my opinion the manuscript does not yet take full advantage of its theoretical analysis. In particular, it does not yet include a detailed comparison of different cell types, neuromodulators or species. Furthermore, the analysis does so far not convincingly show that the fit of the AdEx model or the phase plane analysis provide additional, useful insights as compared to effects of neurotransmitters already identified experimentally (as stated in the manuscript).
    - Following the Reviewer's suggestion, we now use the added complexity to provide actionable insights. The variety of neuromodulatory effects is captured and filtered by clustering the parameters of the model. The excitability landscape we introduced following the Reviewer's suggestion of diving into the bifurcation allows us to simplify the neuromodulatory zoo to two broad families of effects: "switching" or "scaling" the spiking behaviour. This also allows us to connect it to phenomenology (latency, f-I onset, resonance).
    - This also gives us a way to group the effects of a neuromodulator in the following table, whose content guided the redaction of the discussion:

	ACh	NE	HT	DA	5HT
Ctx - human (Fig2)	Switch	Scale	Switch		Switch
Ctx - rodent				Scale	
Striatum (Fig3)	Switch			Scale	
Hippocampus (Fig4)	Switch	Switch	Switch		
TPS (Fig5)	Switch	Switch			
TRN (Fig6)	Scale				
Cerebellum (Fig7)	Switch				Scale

- The manuscript has detailed information on all aspects of the study. I appreciate that the methods are very detailed, but the language used is more appropriate for didactic purposes than for a scientific publication. I would recommend to publish this text as explanation for the github, and aim in the manuscript for a more concise presentation – all methods are standard, and can thus be presented substantially shorter. This allows readers to catch the essential steps more quickly.
    - We thank the reviewer for the comment, we have now shortened the Methods (also removing the unnecessary formulas) and integrated the (already verbose) Jupyter notebooks with some of the removed text.
- Also the presentation of the results uses lots of words. A more concise presentation of the results might profit from a table, in which the results on different cells are summarized. At least it would be helpful to sort effects on neuromodulators by effect shared by all or most neurons, and state those once for all, and a more detailed discussion of where differences occur, and how those could be explained.
    - Thanks to the excitability landscape approach, we shortened the presentation of all results. Please, see the above table used as a guideline in the discussion.
    - Regarding a summary plot where all the different cells and neuromodulators are put in the same space, we tried without a clarity improvement: cell type's parameters and conditions make such that each plot we tried is unreadable. However, in Supplementary Appendix 1, we provide the distribution of the parameters for each condition, mean and standard deviation. This helps the readers appreciate the variability across conditions without losing interpretability, and allows them to compare how each neuromodulator shifts the parameter distributions.
    - For the last point, we rewrote the beginning of the discussion not as a plain summary of the results but to show common similarities and explain differences.
- The authors have decided to fit selected feature of the voltage traces, instead of the voltage traces itself. Could you please give a more detailed explanation of why that was done? Why fitting only six features, instead of the spike time of every spike? The fits that are shown nevertheless overlap largely with the voltage traces, how did you ensure this overlap if the voltage value was normally not fitted?
    - In short, we did so for three reasons: (i) to have a uniform approach to different sources and experimental methods, (ii) use every spike time to constrain every model would lead to over fitting, and (iii) in reality the same neuron does not always respond in the exact same way to the same input. The non deterministic behaviour of neurons is for example showcased in McCormink Williamson., 1989 (a paper that we use to extract cortical neuron voltage traces). Specifically in Figure1 they stimulate the same layer III human cortical neuron from the anterior temporal lobe with the same current protocol and the neuron does not produce identical spike patterns (Figure 1A, B and D) however the overall firing pattern and excitability profile is preserved.
    - Some voltage measures are indeed used to constrain the model and are part of the AdEx itself. This was not clearly written in the text, we have now improved it. Please see also the response to other comments below (L90, L51, L195).

- The conclusion states the results that allowed to "identify the most influential parameters for each neuromodulator.", line 819. Do the authors refer to the components most influential for the PCA dimensions? It would be nice to summarize the findings. A sensitivity analysis of the models could provide additional support for such a statement.
    - Yes, here we specifically refer to the most influential components. For each PCA plot, and each principal component, we report the three most influential components.
    - We agree that a *systematic sensitivity analysis* would support this statement. A full exploration across the 27 conditions (pulling together the different control and neuromodulated traces across brain regions) and 9 parameters, considering that we would need at least 10 variations per parameter and 10 current injection protocols to produce f-I curves, very quickly adds up to more than 24'000 simulations (and this is investigating the effect of 1 parameter at the time). This would be massive in terms of presentation and we feel this goes beyond the main focus of this study.
    We have, as suggested by the reviewer, carried out a targeted sensitivity analysis for the *parameter a* (answer to L292 below), and to ensure transparency and reproducibility we have made the code available. Interested readers/users can extend this analysis to other parameters or conditions when using their own or our data.
- PCAs were done independently for each Figure, which makes comparisons impossible. Could the authors also provide one summary PCA plot in which all control traces are compared with all neuromodulatory traces?
    - We thank the reviewer for this suggestion, which proved very useful.
    Initially, we were concerned that combining all groups into a single PCA would produce an overly crowded plot, but instead it clearly confirmed that brain region identity provides a stronger grouping factor than neuromodulation.

    To illustrate this, we present below two complementary visualizations of the same data: in the first, colors indicate the experimental condition (control vs. different neuromodulators), while in the second, colors indicate the brain region of origin.

    Overall, the data form well-separated clusters, with the exception of the three sets of human cortex models derived from the same cell, which overlap as expected.
    By comparing the two figures we can derive that regional separation is clearer suggesting that brain region identity is stronger than neuromodulatory conditions. The latter however does shift the cells, but these effects remain secondary and do not override the regional identity.
    This fits our intuition: intrinsic cell properties, such as morphologies, ion channel expression and connectivity, are fundamentally constrained by their region, while neuromodulators, despite reshaping the excitability of the cells, do not override those properties.

    It is indeed not surprising that neuromodulation does not manifest in the same way across evolutionarily distinct brain regions.

We have added the second plot to the supplementary material of the manuscript.

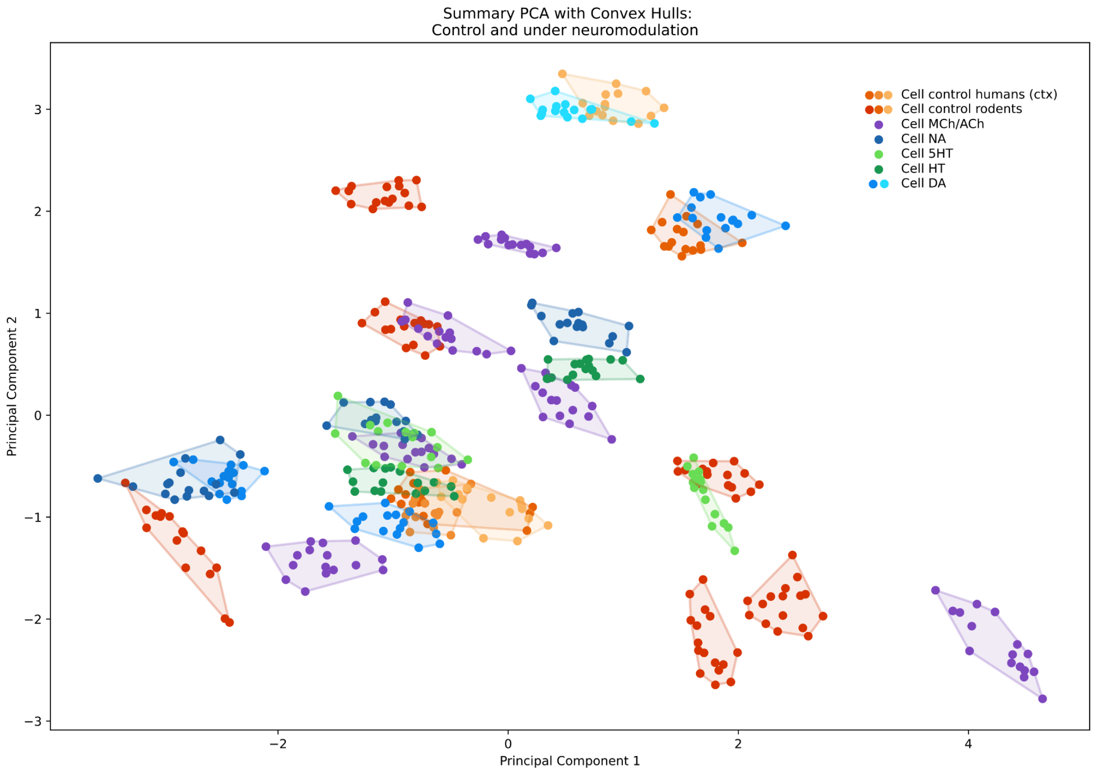

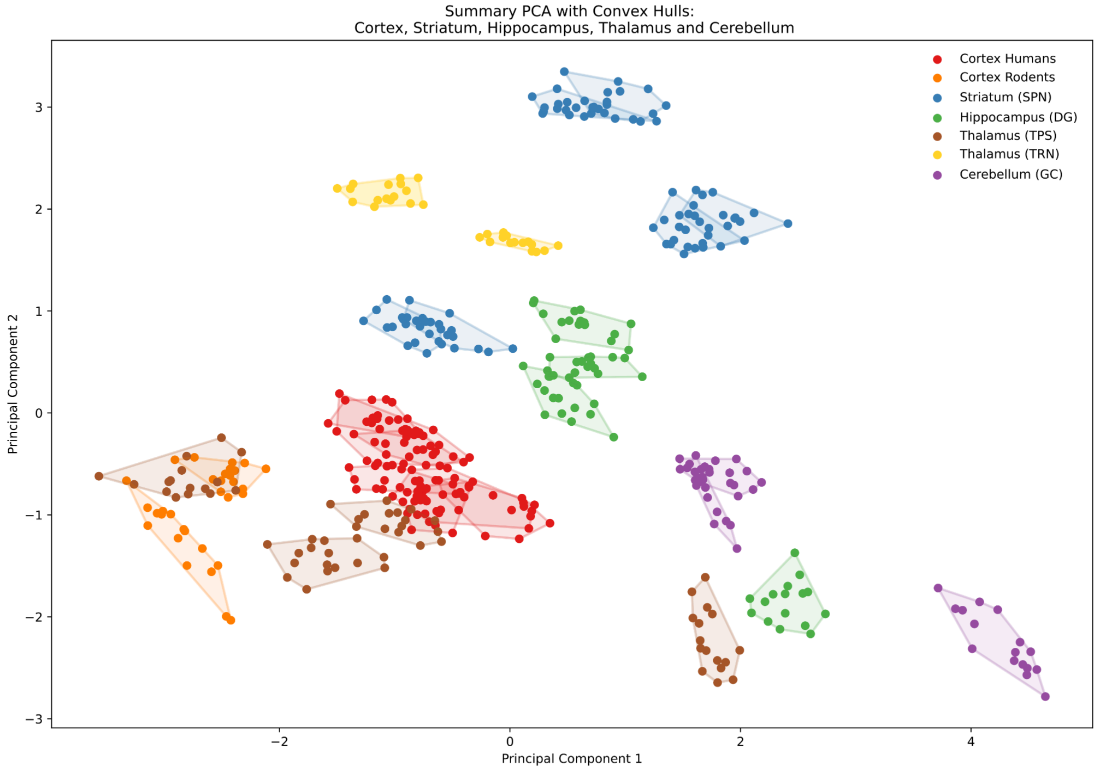

- Do features already fall into different categories? This would be an important question to answer that helps to understand how much more information the AdEx model fit provides as compared to the features space alone. Are changes in feature space and changes in parameter space correlated?

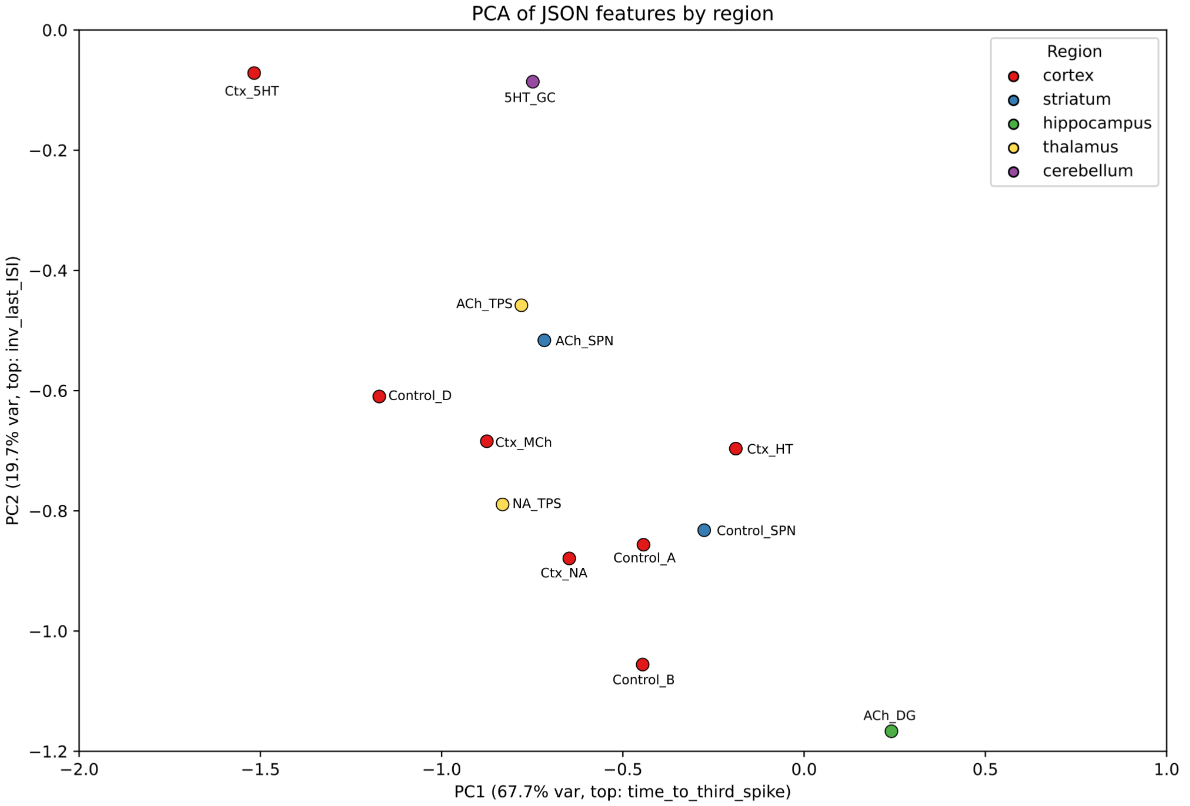

- Thank you for the observation. This is something we thought about but we hadn’t checked because:
  - Performing PCA on the AdEx parameters provides a standardized basis for comparison across models. Parameters are defined consistently for all models, whereas not all the features extracted from traces are always available. For example in the dSPN control the trace has only two spikes, which means that the time to third spike, the time to last spike and the inv to last ISI are not defined.
  - We have only one trace per condition (in most of the cases), so each cluster would contain a single point per condition. In contrast, the AdEx parameter space allows clustering across multiple fitted models, capturing variability and uncertainty more robustly.
  - As noted by the reviewers, some traces express adaptation, making simple metrics like firing frequency less informative for comparison.
- Despite these limitations, we have now performed PCA on the features space (selecting only the condition where all the features could be extracted but excluding the mean firing frequency). What we observed is very interesting. The points corresponding to Ctx_Control_A, Ctx_Control_B and Ctx_Control_D are in this case well separated. Ctx_Control_A is much closer to Ctx_NA, and Control_D is much closer to Ctx_MCh than to the other controls. However, when using the AdEx parameters, the three controls

overlap appropriately (with a negative Silhouette score), reflecting that they correspond to the same cell.

- This demonstrates that PCA in parameter space provides a valid representation of the underlying neuronal identity rather than features alone.
- We have now included the extended features PCA as supplementary figure 6.

- The AdEx neuron model can show different dynamics at spike onset, compare your Ref. [16], https://doi.org/10.1007/s00422-008-0267-4. Have the authors checked which bifurcation type underlies the onset of spiking? This would be a valuable information.
    - We thank the reviewer for pointing this out. In an earlier version of the manuscript we were discussing the bifurcations but we feared it would overload the reader. Now that we have embraced this approach, and the excitability landscape is introduced in the methods, we use the excitability classes to summarize the changes induced by each neuromodulator. This addition has considerably improved the manuscript.
- The paper presents a prediction of neuronal voltage traces for Dopamine. For me this prediction would be only convincing if given a solid motivation. A possible motivation for this prediction could be based on the analysis of the other cells:
    - The construction of the shift in parameters used for this prediction, applied to cells for which the same modulator has been used experimentally, does this correlate with what is observed in the analysis of the other voltage traces? For example, there are four voltage traces in which Acetylcholine is modulated, how do the predicted shifts of those four cases compare?
        - We thank the reviewer for this observation. We kindly ask to read our response to comment L686.
    - You might get clearer results when you fit the modulated voltage traces with a regularization to the control parameters. This allows you to identify the change in parameters that is absolutely critical, in the sense of reached despite the regularization, to reach the modulated state.
        - We thank the reviewer for pointing this out.
          We have not used a “formal regularisation” but when we start the parameter search procedure for the modulated traces we use the same bounds as in the control.
          The Supplementary Appendix, where we show the parameters distribution, the average and standard deviation for each condition should partially address this point.
    - Do modulators change the bifurcation diagram qualitatively?
        - Yes. We now use these changes to introduce the description of results.
- Why do you focus on comparing species instead of, or additional to, brain areas or cell types?
    - We focused only/mainly on comparing brain areas and cell types (please see the attached summary region/cell-type PCA plot). Only for the prediction part we used rodent data to predict DA effects on human neurons.

- Are your results stable when changing the number of model fits that are considered? Doubling the number of simulations and then using the 32 best models, do we end up with the same clusters?
    - Yes. We started with hundreds, we end up getting the same clusters. We reported 16 because of reasonable size to appreciate similarities

<u>Comments in pdf</u>

- *"This work provides a concise framework for understanding neuromodulation, useful for modellers and theoreticians".*
  The paper is mostly about data analysis. Why is this particularly useful for modellers and theoreticians? Would not experimentalists profit even more?
    - We agree that the phrasing may have been misleading, we hope that both computational and experimental communities can benefit from this study.
      The core of the paper is indeed based on data analysis, and our intention is to provide a structured approach to compare neuromodulatory effects across cell types. We aim to facilitate the modelling of neuromodulatory effects in a cohesive way. We have revised the sentence.
- "In this study, we focus on the neuromodulators' ability to alter the excitability and spiking activity of neurons through mechanisms endogenous to the nervous system [5, 7]. (Lines 6-8)". Do the authors refer to intracellular plasticity here? The mechanisms should be better defined.
    - We refer to intrinsic cellular mechanisms such as modulation of ion channel conductances, activation/inhibition of second-messanger pathways, or changes in the membrane properties. We have now added a sentence to clarify this aspect (L 16-19).
- L78: Using this approach we were able to select voltage traces… Please state how many paired voltage traces you have, 15 based on Table 1?
    - We were able to select a total of 30 voltage traces. We have now included the number as suggested.
- L88: Are you only using current clamp traces? Are synaptic currents always blocked in the experiments?
    - We are only using current clamp traces with the exception of thalamic reticular neurons. In this case, the authors of the publication we used as reference applied orthodromic synaptic stimuli (L627).
      To our knowledge, synaptic currents were not systematically blocked. However, since these were slice recordings, the contribution of ongoing synaptic input is expected to be minimal because the slice itself provides a "quiescent environment".
- L90: The features used for spiking are only depending on spike timing, not the voltages (with the exception of the strongly adapting traces). Yet, the fits do map to the voltage of the traces - how is that possible?
    - Excellent observation, we have improved the text to explain this. This text can be found in the manuscript at lines 95-101 and lines 207-210.
      Briefly, the features automatically extracted relate to spike timing, however four features concerning the voltage, such as *holding voltage*, *voltage peak* (V_peak), the voltage corresponding to the initiation of the spike - *voltage threshold* (V_th), and after-spike *reset potential* (V_reset) are manually

measured during the curation of the data once the axes (time and voltage) are calibrated. These values, following reviewer's suggestions, are now included in the metadata file and Supplementary Table 1. Initially only the holding voltage was included.
The V_reset and V_th are used as centers of the parameter search intervals for the corresponding AdEx model parameters. The holding voltage corresponds to the initial value of the voltage and is part of the initial conditions. V_peak is also a parameter of the AdEx and is fixed, all the models of a given condition have the same value.

- L106: Please report the value used for V_th.
  - We now have included this value in the metadata file and in Supplementary Table 1.
- L109: Noise on the trace might lead to the identification of two APs directly one after the other, with just a couple of dt between. Have you made sure that such artificial double spikes did not occur?
  - We did not need to apply any debouncing technique because we didn't have access to any raw recordings and the data we analysed was already "pre-processed". They were quite clean since they had already been used in previous publications to showcase the behavior of specific neurons and the effects of neuromodulators. However, since the number of available traces was unfortunately limited, we manually verified spike detection by plotting the detected spike times as markers over the voltage traces and no unrealistic short ISIs were present.
- L114: How is this measure appropriate for spike adaptation? Would it not make more sense to replace stim duration by the time till the last spike?
  - We agree that in the presence of spike-frequency adaptation using the total stimulus duration may underestimate the firing frequency.
    However, for the purpose of model fitting, our primary concern is not the "absolute value" of the firing rate but rather the consistency of its computation across data and corresponding models. And since this feature is extracted in the same way from both the recorded data and the model simulations, the comparison remains valid. For this reason, we decided to use the same measure across all traces (in the presence or not of adaptation). We have now clarified this point (L 137-144).
- L122: This part could be more concise.
  - As suggested by both Reviewers, to change the didactic approach and move this part to the Jupyter notebooks, we have now removed all definitions for spiking frequency, time-to-N-spike and inverse ISI.
- L129: What is the motivation to restrict this to strongly adapting stimuli? The minimum of spike 2 could for example serve as a meaningful feature. See also comment end of page 3, maybe I missed something?
  - This feature is only extracted in case of strong adaptation because we find that averaging the voltage in the presence of spikes is not representative. However, the after-spike *reset potential* (V_reset) is extracted and used in all the traces. Please, see also the response to L90.
- L140: Why do you use visual inspection instead of the mimimal mean squared error?
  - The heavy lifting filtering is done via the error function based on extracted features which ranks the models. The visual inspection step is just used as a

qualitative check to ensure that the selected models produce spike shapes that are broadly compatible with the experimental traces.
We agree that a fine qualitative comparison, using the mean squared error, would be ideal, but unfortunately the format of the data we have access to doesn't allow for such analysis. The voltage traces were extracted from published figures using WebPlotDigitizer and the resolution of the digitised data won't produce a reliable point-by-point comparison.

- L151: V_peak is used later, where is that defined?
  - Thank you for pointing this out. V_peak was not formally defined and directly introduced directly at L172. It is now defined in the paragraph Data collection and Curation (please, see L 95-105).
- L160: Please explain the motivation for introducing the two classes of parameters. Why calling the second bifurcation parameters although no bifurcations are discussed in this manuscript? Where is this classification used in the paper?
  - We use this classification as it is standard after Touboul and Brette (2008)[1] even though we had not explicitly reported the bifurcation analysis. In the revised manuscript, we now include the bifurcation analysis to study the excitability landscape underlying the control and neuromodulated conditions, together with which bifurcation type underlies the onset of spiking and how neuromodulation affects it.
- L174: The prevents not only spiking, but also makes the neuron ignore any input during that time. Is that the standard approach for AdEx models, or what is the motivation?
  - Yes, the use of a refractory period of a few milliseconds is the standard approach and it's also widely used in simulators such as Brian2 and Nest. Real neurons themselves exhibit a refractory period following an action potential.
- L180: Too much detail in this part.
  - We respectfully disagree with this comment. In the spirit of reproducibility we added in the GitHub repository a requirements file, to be able to have the same software stack. In L180, we only report the Python libraries we used with their corresponding papers.
- L195: What are these user defined ranges here? How where they chosen?
  - For some parameters (for example V_th and V_reset) the ranges were chosen based on the extracted values of these quantities. As mentioned above, the corresponding extracted values are used as centers of the ranges. For the other parameters, the user can either use the default ranges provided (which are intentionally broad to cover a wide variety of electrophysiological behaviours) or constrain them based on prior knowledge and manual pre-tuning.
    For example, if the traces don't show adaptation then the adaptation time constant, *tau_w*, is expected to be between [0, 100] ms.
    In summary:
    - If the user is new to AdEx models, they can use the default values as a starting point and refine them more and more following the output of the

[1] Touboul J, Brette R. Dynamics and bifurcations of the adaptive exponential integrate-and-fire model. *Biol Cybern*. 2008 Oct;99(4-5):319–334.

parameter search procedure;
- If the user has already developed some experience with the AdEx models (which can also simply mean that the user has spent some time trying to manually fit the traces or has used our interactive notebook available on GitHub to investigate the effect of the single parameters of the model) then they can directly refine the ranges based on their intuitions.

- L207,209: Typos
  - Thank you for noticing this, we have corrected the typos.
- L220: This is difficult to understand without a reference. How large was the largest of the minimal error of the fits across voltage traces?
  - The lowest and highest values among all the relative errors are ~ 0.15 and ~4 obtained in correspondence to Thalamic reticular neurons control (Fig 6A) and Thalamocortical projecting neurons control (Fig 5A), respectively. The highest value is an exception and this is due to the bursty and adaptive behaviour of the trace (notoriously difficult to capture).
    The average error value among all the models is ~0.67 and the standard deviation ~0.78. All this information is available on GitHub, if the reviewer considers it important to include in the main text, we will add it.
- L226: How much more?
  - During the parameter exploration phase of those traces we weighted the relative errors in relation to time to last spike and membrane potential at the end of the stimulus up to 5 times more. This helped us to constrain better and better the parameter ranges during early iterations. But once we were satisfied, the weighting factor was reduced to 1.5 to rebalance the contribution of all the features.
    We have now improved the code and made it possible for the user to customize the weighting of individual error components in a user-friendly manner, as we also report in the manuscript (L 237-242).
- L247: Missing.
  - Thank you for noticing this as well. In L247 and other lines there was supposed to be a reference to the S1 Appendix. It is now fixed.
- L274: This is wrong, a saddle point is per definition unstable.
  - Thank you for noticing our poor wording, we corrected it. We meant “If there are both positive and negative eigenvalues, it is a saddle point, exhibiting stable and unstable directions (depending on the sign of the eigenvalue)”.
- L280: What is the motivation for the absolute value? Are there cases where the parameters switch sign between mod and control?
  - Yes, in some cases (see Appendix 1) there is a switch in the sign of the parameter *a*, for example in the direct striatal projection neurons (control vs DA), dentate gyrus neurons (control vs ACh-NA), and thalamocortical projecting neurons (control vs Ox).
- L290: This does not make sense to me. In S4, the predicted parameter "a" switches sign as compared to control because the ratio becomes negative. This seems a bit extreme, better could be avg_human_control_j*(1-ratio_j)
  - We are not sure we understand the suggestion.
    Avg_human_control_j * (1-ratio_j) = Avg_human_control_j * ratio_i
    And hence we are back to the first case (*absolute value strategy*).

To explain with a concrete example the rationale behind the *signed value strategy*, let us do a back-of-the-envelope calculation.

The avg_rodent_control_a = -0.007 and the avg_rodent_mod_a = -0.01, so the value of *a* decreases under modulation, and hence:
ratio = avg_rodent_mod_a / avg_rodent_control_a = 1.43.
The avg_human_control_a = 0.0045 and if we apply this logic/proportion:

Avg_human_mod_a : avg_human_control_a = avg_rodent_mod_a : avg_rodent_control_a,

we get to:
Avg_human_mod_a = avg_human_control_a * avg_rodent_mod_a / avg_rodent_control_a
Which means:
*Avg_human_mod_a = 0.0045 * 1.43 = 0.0064 ← a1.*

So in the human case, the value of *a* under modulation has increased (from 0.0045 to 0.0064), while the value of *a* under modulation in rodents had decreased (from -0.007 to -0.01).
And this is what we have in the *absolute value strategy*.

However, if we want the parameter a to decrease and we want to take into account the possibility that it changes sign, instead of multiplying the avg_human_control_a value by 1.43 we multiply it by -0.43 (which correspond to 1 - avg_rodent_mod_a / avg_rodent_control_a). So in this case we get:
*Avg_human_mod_a = 0.0045 * (-0.43) = - 0.00193 ← a2.*

These two strategies are "the most extreme". For example other two possible ways consists in using as scaling parameter:
-- (avg_rodent_mod_a / avg_rodent_control_a - 1), which might be what the reviewer suggested. In this case the *a* value is decreased to 43% of it value
*Avg_human_mod_a = 0.0045 * (0.43) = 0.00193 ← a3*;
-- (avg_rodent_mod_a - avg_rodent_control_a + avg_human_control_a). In this case we ensure that the slope of the decrease is the same in the rodent and human models
Avg_human_mod_a = -0.01+0.007+0.0045 = - 0.0015 ← a4.

In the plot below, which we also add to the S1 Appendix, these values are represented. We have expanded on this in *Materials and method - Predicting the effect of a neuromodulator* and in Supplementary figure 4.

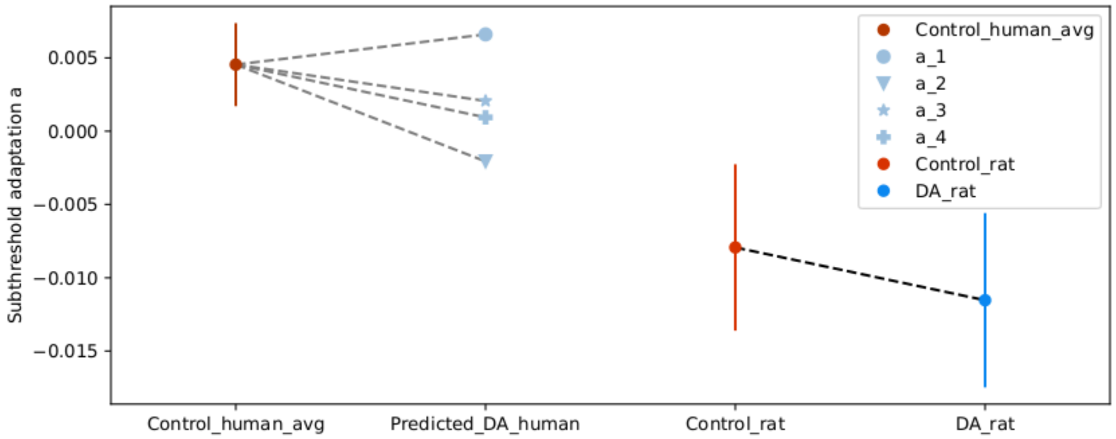

- L292: How sensitive is the fI curve to this parameter? -> sensitivity analysis.
  - We thank the reviewer for this suggestion, we have now included the sensitivity analysis of the parameter *a*. The results are added to the Supplementary Figure 4.
    Briefly, changing the parameter *a* within the range determined by the two strategies reported in the text does not drastically alter the f-I curves and its effect is more pronounced at lower current injections. Indeed the standard deviation of the firing frequency across different values of *a* decreases from about 5Hz at low current to about 2 Hz at high current injections. This is comparable to the intrinsic variability experimentally observed within a given cell type.

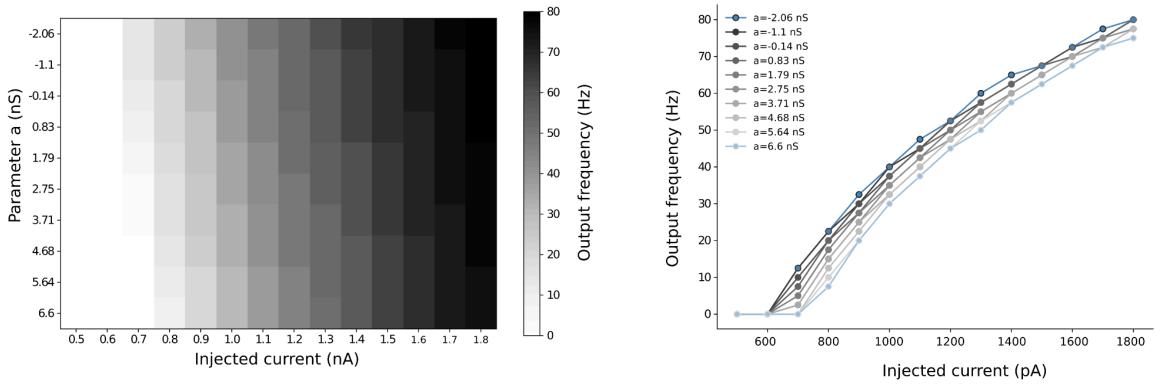

- L319: How do these features compare to other features used in the literature? Why not fitting the spike timing of each and every spike?
  - In Naud et at., 2008, for example, firing rate, time to first and second spike are used. We have used more features to improve the description of the voltage trace while keeping the computational cost very low.
    As mentioned above, taking into account the spike timing of each and every spike would lead to overfitting because the same (real) neuron does not respond identically to repeated current injections of the same magnitude. This

can be for example seen in McCormink Williamson., 1989. The panels A, B and D of their Figure 1 show the membrane potential of the same neuron when stimulated with the same protocol and the neuron does not respond with an identical spike pattern but the overall firing pattern and excitability profile is preserved.

- L340: How many models were simulated?
  - Because Sobol sequences are built recursively using binary representations, for each simulation we generated 2^n models, with n varying from 10 to 16. Since our pipeline runs in parallel, even for 2^{14} models, it takes only around 3 minutes to complete. For each cell type and condition multiple simulations are needed, depending on the suitability of the initial parameter bounds.
- L371: You here talk about the approach of the threshold from above, after the neuron started spiking, don't you? Approach of the threshold can be misunderstood as the initiation of spiking.
  - It is the initiation of spiking. We give here how a neuromodulator alters the dynamics.
- L381: All phase planes are plotted at some I that ensures spiking?
  - Yes. The starting point is the resting membrane potential, to look at the dynamics of approach to threshold.
- L395: What are different excitability states over time?
  - The sentence was indeed a little ambiguous, what we mean is that: "Combination of these 'leak' permeabilities, gL (in the AdEx), provide a mechanism by which neuromodulators can dynamically shift excitability regimes as their receptors are activated over time".
- L396: You here explain the effect on the phase plane by the underlying parameters. But what is the gain of knowing the phase plane? Bifurcation analysis could be the answer.
  - We take the opportunity to thank the reviewer again for this suggestion. We changed the whole result section by adding how neuromodulators cause the bifurcation of each cell's behaviour.
- L406: Was there anything shared? What is the understanding gained?
  - As stated above, and now throughout the new version of the paper, using our framework (feature extraction, model fitting, clustering, and excitability landscape) we learn that we can reduce all neuromodulators to two families of effects, either switching or scaling the spiking behaviour.
- L419: Is this typically allowed in the AdEx? What happens if you constrain the fit?
  - Yes, it is. For example in Naud et al., (2008, *Firing patterns in the adaptive exponential integrate-and-fire model*), a widely cited paper, the authors presented AdEx models of eight distinct firing patterns and in four of these cases the parameter *a* has negative values.
- L422: What do we learn from this analysis that is not yet known experimentally?
  - Same answer as L406.
- L426: "the slopes of V- and w-nullclines" can be better understood
  - If the left branch of the V-nullcline is less steep than the control case, this means that the neuron requires less current to destabilize the resting state and move toward spiking. We can say that the resting state becomes more sensitive to small perturbations.

- The slope of the w-nullcline encodes the parameter a (adaptation rate). When the adaptation is weaker and hence the slope is lower, once the neuron starts firing there is less negative feedback to counteract depolarisation. This is why lower slope means higher excitability.
  - Moreover, when looking at more depolarised membrane potentials, if the intersections between nullclines shift to the left, and hence the distance to the bifurcation point (where the neuron transitions to spiking) is reduced, the cell becomes closer to threshold and therefore more excitable.
  - These effects shift the neuron toward easier spike initiation and more persistent firing.
  - We have included this text in the manuscript.
- L427: Where is V_peak defined?
  - Same answer as L151.
- L429: The presentation here is just a report of changed values, please add more relation and understanding to the presentation.
  - We have now added more insights thanks to the bifurcation analysis.
- L444: Do you find also an increase in the slope before the spike in the original voltage traces, not the simulations?
  - The steeper slope is measured in both the original voltage traces and simulations.
- L468: Animal model or mathematical model?
  - In rat animal model, we have now specified this in the text.
- L529: Why not use the same colours for all subpanels? Are the colours color-blind friendly? Red and green should not be used without taking this into account.
  - Thank you for noticing this. There is indeed an error in the text; the color in the panels is the same. We have now fixed the text.
    In general, we used the same colour scheme across subpanels: same colours were assigned to the same conditions, while related colors (for example red, red-orange, orange) were used to distinguish species or receptor subtypes (for example humans vs rodents or D1 vs D2 striatal neurons).

    It is a "very intricate colour palette":
    color_palette = {"control_I": '#e66101', control_II": '#f28d32', "control_III": '#fdb863', "control_rodent": '#da3500', "control_avg": "#b63800", "control_d1": '#e66101', "control_d2" : '#fdb863', "control_SPN": "#da3500", "HT": '#1a9850', "ACh": '#7f46c2', "NA": '#2166ac', "5HT": '#6ce058', "DA": '#9bc1e0', "DA_human": '#9bc1e0', "DA_rodent": '#0c89f0', "d1_DA": "#0c89f0", "d2_DA": "#23e0ff"}

  We also wanted to be as color-blind friendly as possible. We consulted, for example, https://colorbrewer2.org, but we soon realised that the recommended "sequential colour scales" were not sufficiently distinguishable for the number of categories we needed, and instead we opted for this palette, allowing a clear visual separation between groups.

- L667: Why not changing the parameter "C"? Is C fitted or assumed fix?
  - The parameter C is modelled and varies between cell types.

- L686: This needs a solid motivation based on predictions for "known" voltage traces, see main comments.
    - We thank the reviewer for this observation. We agree that it would be nice to use the “prediction strategy” in cases that we know to validate the method.
    Our assumption for this prediction strategy is that the slopes of the control f-I curves (of the cortical human and rodent neurons, in our case) are reasonably similar. Unfortunately, we were not able to find datasets where the control neurons display comparable f-I slopes (across brain regions or across other species), which would have allowed us to directly test the strategy.
    Our rationale is that if neurons of the same brain region display comparable intrinsic excitability across species, then predictions of the effect of a given neuromodulator are “more likely to transfer”. This reasoning was actually strengthened by the PCA summary analysis that the reviewer suggested, since it revealed a stronger clustering based on brain regions rather than neuromodulators.
    We examined the control models from different brain regions and, as the plot below shows, we found pronounced differences in the f-I slopes. For this reason, we could not validate this strategy; however, we will continue to search for suitable experimental data to test this approach in future work.

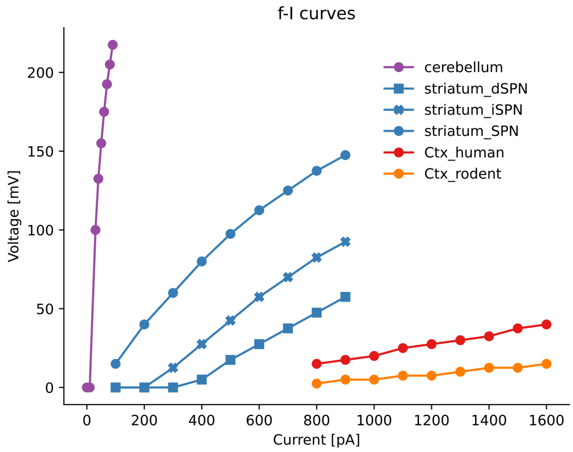

    As the reviewer can imagine, we did not include the control traces of neurons in hippocampus and thalamus since they exhibit adaptation.
- L754: Are the parameter bounds and the start intervals the same? Where are they reported?
    - They are not the same. Usually, after a few iterations the parameter bounds can be tweaked and this allows us to focus the search on more plausible parameter ranges, improve the fitting efficiency and make better use of computational time. However, increasing the number of samples (and hence models) and keeping the starting intervals unchanged would give the same results but using more computational power. The parameters are reported on GitHub.
- L760: This is interesting, have you found any such differences? The phase plots shown do not include any of these features, do they?

    - We did not encounter such behaviours; we chose to keep the phrasing general to highlight the potential of the approach, though in this specific study the dynamics remained within the tonic-bursting spectrum.
- L772: How is that meant, to me it seems to not fit in a scientific paper.
    - We respectfully disagree, but we should have expanded on the reason why we decided to write our AdEx solver in Python.
      Brian2 and NEST are powerful tools designed for large-scale network simulations, they introduce heavy dependencies and additional unnecessary syntax to learn. Instead, using Python directly and writing our own AdEx solver we make the workflow more explicit and accessible to researchers independently of their expertise.
- L777: Where do I see this? E_L is not mentioned in the results, is it?
    - Thank you for this comment. E_L was introduced at line 162 and mentioned here and there, but now in “Materials and method” we have expanded on this and reported the different values in the Supplementary Table 1 (together with other extracted voltage-related parameters, as mentioned above L90).
- L804: I am missing the compact descriptions.
    - Please, see how the excitability landscape reduces different neuromodulators to two families.
- L820: Only steady states where discussed, where should I see the effect on neuronal dynamics?
    - We heavily changed the discussion of dynamics, thanks to the new excitability landscape approach, and also added streamlined phase plots to render more explicitly how different conditions affect the dynamics.
