# Supplementary material for: A unified model library maps how neuromodulation reshapes the excitability landscape of neurons across the brain
Source: PLoS Comput Biol. 2025 Dec 1;21(12):e1013765. doi: 10.1371/journal.pcbi.1013765 (PMC12680334; doi:10.1371/journal.pcbi.1013765)
Supplement: Attachment — Submitted filename: remarks_to_authors_2.pdf [file pcbi.1013765.s013.pdf]

I would like to thank the authors for their detailed and helpful response to my feedback. The GitHub repo is thorough and easy to navigate, the excitability landscape and streamlines added to the figures are nice additions, and all of my more minor points have been sufficiently addressed. I have responded to a few small points below, but these comments should be taken purely as suggestions that do not require further review from me.

Paper title

If I had to vote for one, I think it would be #1. I like that it emphasizes the model library, which is a major contribution, and the fact that the work covers multiple brain regions. It also communicates that the work is focused on neuromodulatory effects *on neuronal excitability* as opposed to some higher-level construct (population geometry, fMRI representations, etc). I have to admit that "switching or scaling" is very catchy, though.

Euler time step size

0.01ms is certainly more than fine enough. The authors could add "using a default time step of 0.01ms" to line 181.

Additional reporting on stimulation duration and temperature dependent

I would like to thank the authors for tracking down and documenting these experimental details, which will make their library easier to reuse.

Current used to calculate voltage nullclines

> It seems like the level of input current used to calculate the voltage nullclines isn't reported. I assume zero, but this would mean that the phase plots correspond to a state where the neuron isn't spiking (consistent with the presence of a stable fixed point at resting membrane potential), unlike the sample traces. I found this discrepancy a bit confusing.
>
> > The input current used to calculate the nullclines is not zero; it varies across cells, and corresponds to the values reported in the referenced articles. These details are provided in the Jupyter notebooks, and we chose not to include them in the main text to keep it concise. As a result, the phase plots do not correspond to a pure resting state. Instead, they show all possible states, with the resting state appearing as one attractor fixed point, and they capture the full range of voltage dynamics, including the sample traces. We wonder whether the reviewer's comment reflects a misunderstanding of our explanation, or whether we misunderstood the reviewer's comment.

I think perhaps both. My confusion came from the fact that the sample traces show a baseline period of quiescence followed by a step input that drives spiking and then a return to baseline, and I would expect the V nullcline to lie above the w nullcline during the step period when spiking actually occurs. It was unclear to me which input level the V nullclines on the plot represent, but they did not seem to correspond to the step period. Based on the authors' response, I take it that the V nullclines shown correspond to the holding current (baseline); perhaps a couple of words could be added to line 421 or the figure caption to make this explicit. I would also suggest removing the black traces on the phase plots in Fig. 1 to eliminate any discrepancy between the spiking shown in the trace and the location of the V nullcline, to reduce visual clutter, and to improve consistency with the insets in later figures.

Bayesian-adjacent approach

> L753-756: The general approach of using prior information to constrain the parameter search space and reporting a distribution of models rather than a single best-fitting option feels a bit Bayesian in spirit. Perhaps the authors could comment on this?

This is an interesting point, we did not consider. Our optimisation starts with biologically informed parameter ranges (a de-facto prior), then samples that space quasi-uniformly (Sobol) and retains the parameter sets whose simulated features fall within a tolerance of the experimental data. The resulting ensemble behaves much like an approximate posterior: it encodes parameter uncertainty and lets us propagate that uncertainty to any derived quantity (f-I curves, phase-plane structure, etc.). Although we stop short of a fully Bayesian treatment (there is no likelihood function or MCMC), our procedure shares two Bayesian aspects:

- Prior knowledge constrains exploration. Bounds taken from the literature keep the search focused on physiologically plausible regions, just as an explicit prior would.
- Inference yields a distribution, not a point estimate. Rather than collapsing to a single 'best' model, we report the entire cloud of admissible solutions, analogous to drawing samples from a posterior.

A stricter Bayesian formalism, our workflow could be reframed as an Approximate Bayesian Computation (ABC) scheme:

- the Sobol sequence becomes the ABC proposal step;

- the feature-space error is the ABC distance metric;

- the acceptance threshold controls posterior width.

Moving to a full ABC-SMC or MCMC implementation would require: defining an explicit likelihood or kernel, and updating the sampling strategy. We chose the current approach for computational efficiency and ease of interpretation, but the code base is modular and could accommodate a more traditional Bayesian loop if future work calls for it.

The authors' response is insightful. I would add that the weighted absolute percent error metric seems to imply a Laplace (log) likelihood with covariance determined by the feature weights and magnitudes. Under a uniform prior, the posterior would be proportional to this likelihood. With that in mind, the parameters of the selected models seem to form something almost like a rough $16/N_{\text{draws}}$ credibility interval, except that the retained parameters are not weighted by the likelihood as a posterior would be. If a user of this library eventually decides to use a squared error metric and weight the retained models according to relative accuracy, this becomes quite close to Bayesian estimation. I would suggest that the authors briefly mention these similarities and differences in the discussion so that downstream users realize that Bayesian tools may be useful if such modifications are needed, but, as stated above, this is completely optional.

Implications for brain disorders

The authors discuss the relationship between disordered neuromodulation and psychiatric disease on lines 873-878. While the cause of Parkinson's disease is clear-cut, not everyone would agree that serotonergic and/or noradrenergic dysfunction contributes to depression. See the controversial review of Moncrieff et al. (Molecular Psychiatry, 2023) and response by Juahar et al. (Molecular Psychiatry, 2023), for example. I would suggest either inserting some weasel words ("thought to contribute" vs "contribute") or focusing on the prominent role of drugs that target neuromodulator systems in psychiatric treatment (SSRIs for depression, 5-HT2A and D2 antagonists for psychosis, dopaminergic stimulants for ADHD, etc).
