# Supplementary material for: A unified model library maps how neuromodulation reshapes the excitability landscape of neurons across the brain
Source: PLoS Comput Biol. 2025 Dec 1;21(12):e1013765. doi: 10.1371/journal.pcbi.1013765 (PMC12680334; doi:10.1371/journal.pcbi.1013765)
Supplement: Attachment — Submitted filename: Response_to_Reviewers.pdf [file pcbi.1013765.s014.pdf]

PCOMPBIOL-D-25-01115 - Revision 2

Answers to Reviewers' comments

We would like to thank the reviewers for their continuous effort in helping us better shape the manuscript.
We refer to the first revision of the manuscript (with track changes) for the line numbers.

Reviewer 1:

- Paper title: Option #1? We have indeed selected option #1. Thank you for having made us reflect on this.
- Euler time step size: Thank you for the suggestion, we added "using a default time step of 0.01ms".
- Current used to calculate voltage nullclines:
  - couple of words could be added to line 421: "V nullclines shown correspond to the holding current".
  - We apologise for the confusion we caused. The reviewer is indeed right and the nullclines are computed in correspondence with the baseline holding current. We have explicitly written this in the text now. The python function we implemented to compute the nullclines takes as one of the input such current and can be changed by the used to plot the nullclines for any other preferred value.
  - I would also suggest removing the black traces on the phase plots in Fig. 1: Thank you for the suggestion, we agree that it improves consistency with the other figures.
- Bayesian-adjacent approach:
  We thank the reviewer for making us reflect on this aspect. We have added the following text in the discussion:
  "Our approach shares conceptual similarities with Bayesian inference. The weighted absolute percent error we use as an objective function can be interpreted as corresponding to a Laplace log-likelihood, where the feature-specific weights implicitly define the covariance structure. Under a uniform prior, this would make the posterior proportional to that likelihood. In our current implementation, however, the retained parameter sets are treated as equally plausible rather than being weighted by their likelihood, so the resulting distribution resembles an unweighted approximation of the posterior. Using a squared error metric and weighting models by their relative accuracy would bring the procedure even closer to a Bayesian estimation scheme."
- Implication for brain disorders: either
  - inserting some weasel words ("thought to contribute" vs "contribute")
  - focusing on the prominent role of drugs that target neuromodulator systems in psychiatric treatment (SSRIs for depression, 5-HT2A and D2 antagonists for psychosis, dopaminergic stimulants for ADHD, etc).
  - Thank you for the suggestion and for the articles. We have both rephrased and added the reviewer's suggestion on the psychiatric treatments.

Reviewer 2 (comments from the email):

Major:

1. *Please review the manuscript and change all mentioning of "class A" and "class B" with "class 1" and "class 2" - or provide a definition/reference.*
   - As suggested, we replaced all class A and B to class 1 and 2, respectively.
2. *The advantage of fitting voltage traces is that we translate voltage differences into parameters that have a biological meaning. Yet, I am still missing the interpretation of the results with regard to a reasonable biological action of the different neuromodulators. For many of them, mechanistic points of action are known, e.g. how they change the resting potential, how do these known mechanisms fit to the biological picture the parameter changes are drawing?*
   - This question prompted a rewriting of the reference discussion included in several result sections. We thank the reviewer for it. We did not make sufficiently clear that the declared goal of this work is to reach a comprehensive understanding of what neuromodulation means for the dynamical properties of neurons. To achieve this goal, we have fitted the voltage trace of different neurons from different areas under different neuromodulators to the same model with its parameters. We deliberately chose a model whose parameters have a degree of biological meaning. And this gave us the ability to provide better insights. Please see the revised version for all the changes we made.

3. Section "Predicting the effect of a neuromodulator": The authors did not provide evidence that what they call prediction has indeed predictive power. I recommend that the authors relabel this subsection and more carefully talk about "a proof of principle that this approach could predict modulatory effects given sufficient data". Generally, the focus of the paper has shifted away from the prediction, which is also reflected in the title, which is good.
   - We agree and thank the reviewer for the suggestion. We have relabeled the subsection (“A proof of principle: estimating the effect of Dopamine on Human Cortical pyramidal neurons”), we have improved the text of this section and carefully substituted “prediction” with “estimation” in all the other parts.
     We would also like to kindly clarify that the focus of the paper has never meant to be a creation of a tool to predict the effect of neuromodulators per-se. Rather, while fitting the different data traces we realized that this approach could, in principle, be extended toward such applications, and therefore decided to include this analysis in the manuscript. We hope that, in the near future, additional experimental data will allow us to validate and further refine this approach. In that eventuality, we will add an additional Jupyter notebook with the analysis.
4. The authors state that a prediction of neuromodulation is only possible with similar baseline excitabilities, and hence refrain from additional examples. I find this a weak excuse, as I do not agree with the authors that the cortex human and rodent baseline excitabilities are more similar as compared to striatum iSPN/SPN. The f-I curve is more similar in the latter case when normalizing by the highest value, which would in my opinion be a fair comparison.

- ○ We respectfully disagree with the reviewer. We have appreciated the reviewer's comment about looking for ways to validate the approach (during the first revision), and as already mentioned this is something we had thought about and we put a lot of effort looking for a dataset that we could use as a validation tool. Unfortunately we haven't been able to find one so far.
  In light of this impossibility to validate our approach, we have now changed the wording and used "estimation" instead of "prediction". We hope that the reviewer agrees with this change.
  About the normalization suggested by the Reviewer in this revision, we have done it and the results clearly show that the cortex human and rodent baseline excitabilities are more similar as compared to striatum iSPN/SPN:

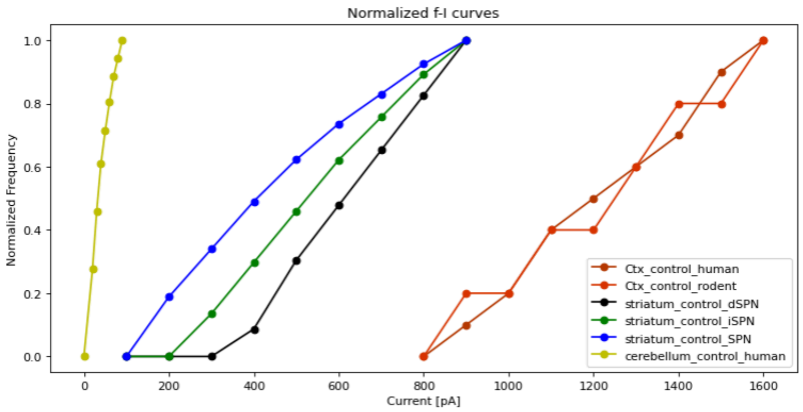

- ○ We appreciate the suggestion of comparing iSPN and SPN, however we don't have access to studies in which iSPNs and SPNs are "separately subject to the same neuromodulator". We could find data for dSPN-iSPN with and without DA bath application and generic SPNs with and without ACh bath application.

Minor:

- Thank you for the PCA plots including all models. It might be interesting to add arrows that link the center of the untreated case with the treated case to see not only the clustering -which is not evident- but also a systematic shift along the PCA components -- similar as done for the excitability landscape plots, but now for all at the same time. For example, Cerebellum and Thalamus (TRN) neurons (and mayby striatum?) react to MCh/ACh with a right downward shift along the principle components, while Thalamus (TRS) neurons show a shift in the opposite direction - any idea how to explain this? Different baseline features? Given the new Figures S5 and S6, if you connect control and treated locations in PCA space with an arrow, do you see more ordered responses in S5 as compared to S6? This is what you suggest, given that models are a more encompassing account of what happens under neuromodulation?

- We have added the arrows and each arrowhead style represents a neuromodulator.

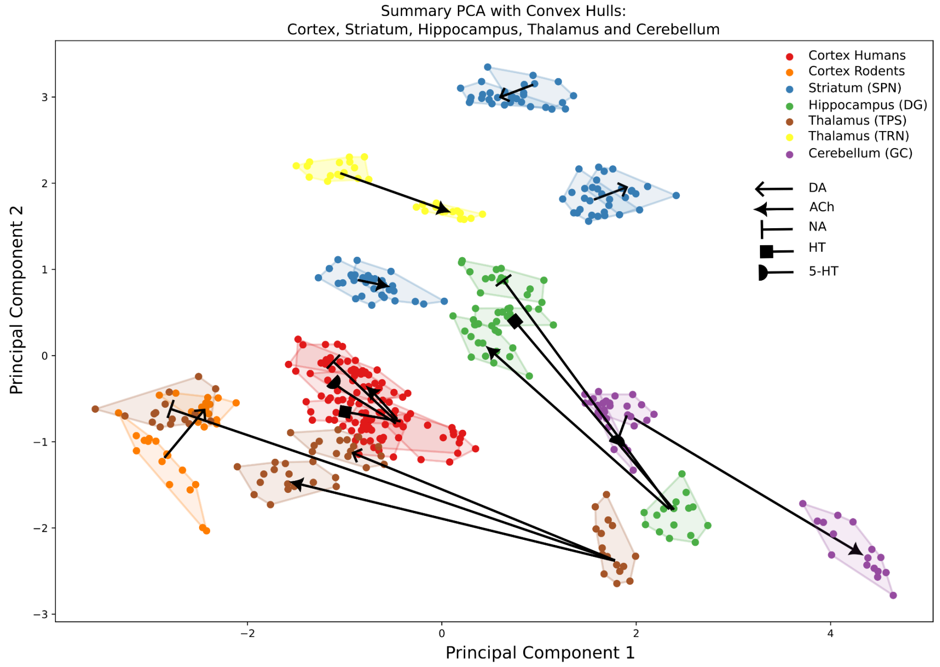

- We thank you for this observation and to comment on this we would like to expand it and include also the hippocampal neurons. In the TPS and hippocampal neuron case the "ACh arrows" are left upward. We have noticed that hippocampal neurons and TPS neurons share more similarities than for example TPS neurons and TRN neurons despite these two belonging to the same brain region.
- This seemed controversial to us too. However, after searching the literature, we found that, for developmental reasons, hippocampal and TPS neurons are more similar to each other than TRN neurons are to either of them. Despite their very different adult functions, the hippocampus and thalamus both arise from the embryonic forebrain (prosencephalon) and thus originate from the alar (dorsal) regions of the neural tube [embriology]. At the same time, the reticular formation is largely a product of the hindbrain (rhombencephalon) and partly the midbrain, not the forebrain [frontiers].
- About the comparison between S5 (parameter space) and S6 (feature space), in our opinion the key interpretation is the following: in the parameter space (and hence following the models), the "groups" that we know a priori to belong together (for example, the three control cortical models) are clustered, while when looking at the features space they are not only further apart but also closer to different groups than to each other. And in this sense the model is a "better lens through which to view the data" than the features. More formally, the parameter space captures the neuronal identity more accurately than the features alone.

- My request for a sensitivity analysis was not sufficiently clear to be understandable. The authors addressed the request by a targeted sensitivity analysis for the parameter a and concluded that within the relevant range of a values, the changes to

the model are only quantitative. I would find it more interesting to compare how strongly parameter a changes the model as compared to other parameters. A large change in parameter a might result in the same effect as a much smaller change in a second parameter, but PCA only identifies the parameters with the strongest change, not the strongest impact on selected/relevant model readouts. Here you could generate additional insights and take advantage of the model.

- We understand the idea behind the sensitivity analysis, however, as mentioned, a full exploration across the 27 conditions (pulling together the different control and neuromodulated traces across brain regions) and 9 parameters, considering that we would need at least 10 variations per parameter and 10 current injection protocols to produce f-I curves, very quickly adds up to more than 24'000 simulations (and this is investigating the effect of 1 parameter at the time). In addition, once the parameter scan has been done for this amount of combinations, one needs to inspect all plots, choose new plots to merge the information and so on. This would be an entirely new study.
  We provided (on the GitHub folder) the code to run this analysis one parameter at the time (since we did it for the parameter *a*) so that interested readers/users can perform it on smaller datasets maybe focusing on others parameters or conditions when using their own or our data.

- Switching and scaling is an interesting distinction to make, and the table shown in the rebuttal letter would be a nice addition to the manuscript. The transition between tonic and bursting is a second category that the authors could consider in this table, as even stated as example in the discussion.
  - Following your comment at line (L953), we added the cautionary note (on L1014) that we can only roughly map tonic and bursting onto class I and 2. It would be interesting to look at "tonic-ity" and "burst-icity" through the lens of switching and scaling, but in this work we wanted to understand the role of neuromodulators on the dynamic. Trying to use this method to settle the long-standing debate over tonic vs bursting (which led to the better approach of class I and II) we fear would go beyond the scope of this study.

I have a few more comments in the appended pdf, which the authors could address if they wanted to, but which are not prerequisite for recommending publication.

Reviewer 2 (comments from the pdf which the authors could address if they wanted to, but which are not prerequisite for recommending publication):

1. L20: you do not relate the parameter changes to these intrinsic mechanisms, thus it sounds wired to me to state "we focus on..."
   - Thank you for the suggestion. We updated the text.
     i. Here, we study how neuromodulation influences the dynamical properties of neurons. Neuromodulators act through various endogenous mechanisms, such as the modulation of ion channel conductances, the activation or inhibition of second-messenger

pathways, and alterations in membrane properties [5,7], creating a cascade of interpretability problems that motivated this study.

2. L204: Please define V_peak here
   - Thank you for the suggestion. We updated the text.
3. L244: ... are fixed by the voltage traces.
   - Thank you for the suggestion. We updated the text.
4. L246: Please consider whether it would be good to separate the description of your tool from the description of what you did to get your results. Here they are mixed up.
   - Thank you for the suggestion. We updated the text.
5. L275: Is the 9 and then 1.5 as weight stated somewhere in the Supplement?
   - The parameters to explore (9 in the “standard case”) and the weight of the features are reported in the GitHub. What we mean by “standard case” is that the user can also change that (in addition to the weights). Let’s say, for example, that a user wants to use our tool but also wants to take into account a parameter from another publication and hence decides to have it fixed to such a value, our tool allows for such modularity.
6. L338: but? Grammar?
   - Thanks for pointing out this typo. We changed to: “or scale the onset bifurcation ~~but~~ *by* modulating its expression”.
7. L355: Would it be worth to quantify the distance to spike threshold? Does this intuition correlated with threshold current in the fitted model?
   - In principle, yes. However, it would require additional dynamical analyses and systematic explorations to map how perturbations evolve in the vicinity of threshold, essentially, a study of the attractor basin. The qualitative intuition is consistent with the results, and we consider this an interesting direction for future work

8. L367: Why do the baseline excitabilities have to be comparable? Would the same excitability class not suffice?
   - When we did the "prediction/estimation analysis" we hadn't done the excitability class analysis yet. And back then our rationale was that if neurons of the same brain region display comparable intrinsic excitability across species, then predictions of the effect of a given neuromodulator are “more likely to transfer”.
     When we added the excitability class analysis we thought about it but then we noticed that for example while rodent and human cortical control neurons exhibit comparable intrinsic excitability they belong to Class I and II, respectively (Fig. 8E).

9. L388: Where is that shown? Refer to Supplement or state not shown.
   - Thank you for the suggestion. We added a reference to the Appendix and the Jupyter Notebook.
10. L429: Is this minimal in a strict sense? If you remove any observable, the fit becomes worse?
    - These features are “minimal” in the sense that they are few in number and can be obtained from virtually any published electrophysiological recording, even when, as in our case, tools such as WebPlotDigitizer are required to extract traces from figures. Nevertheless, we verified that arbitrarily removing

observables does not prevent the fitting procedure from reaching low error values, although it may require more sampling and hence computational time.

11. L456:
    - Thank you for the suggestion. We updated the text.
12. L486: Here you should make clear that you are now jumping to one particular neuron type. Do you recognize some logic behind the grouping of the dominant PCA components?
    - Thank you for the suggestion. We updated the text:
      "When focusing on a specific neuron type, in this case cortical pyramidal neurons (Fig. 1), it is possible to assess the consistency of this approach and examine the insights it may provide."

13. L500: Could you summarize these insights into one sentence before going into details?
    - Thank you for the suggestion. We updated the text.

14. L591: How do these PCA comp. compare to the ones from Fig.1? What information should the reader take from this statement?
    - In Fig. 1 we reported, for clarity, the PCA and the excitability landscape only the control and one neuromodulator. In Fig. 2, we apply "in full" our method and we can see how the parameter space (Fig. 2E) correlates with the excitability landscape (Fig. 2F). These two spaces are inherently different. The PCA shows how parameter values group in different conditions. The Excitability landscape shows how the dynamic of neurons changes in different conditions.
15. L953: switching between burst and tonic is not 1-to-1 related to switching between type I and II. You here consider the latter, I assume?
    - Thank you for the suggestion. We updated the text.

16. L952: Why is Fig. 3 Acetylcholine classified as switching and Fig. 6 not? For Fig. 6 the location of the control seems to be on both sides of the separatrix? Same as for Fig.3 the modulated models.
    - Thank you for the comment, we take this opportunity to further clarify this point in the answer below.

17. L955: Interestingly, switching only occurs when controls are below the separatrix...
    - This is a very interesting fact that we noticed but did not comment on. Thanks for bringing us to expand and provide this additional insight.
    - This has to do with the relationship between *a*, the subthreshold adaptation conductance, *C_m* the membrane capacitance and *tau_w*, the adaptive time constant.
    - The Bogdan/Takens boundary is defined by *R_a=R_tau*, that is where *a/gL* = *tau_m/tau_w* and considering that *tau_m* = *C_m/gL* this becomes where *a* = *C_m/tau_w*. In other words, *gL* canceled out.
    - So, the excitability class depends only on the relation between *a*, *tau_m* and *C_m*. Class I corresponds to the models where *C_m* > *a*tau_m* while Class II to the models where *C_m* < *a*tau_m*.
    - Often, neuromodulators reduce *a* and/or *tau_w*.

- For example ACh (via M-current suppression), NA/5HT (via closing K+ leak or speeding adaptation kinetics) decrease *a*tau_w*, pushing the system toward/into the Class I.
- Many modulatory actions also change *gL*. But varying *gL* scales both coordinates the same way and the points move roughly along the Bogdan/Takens boundary, which does not alter *a*tau_w* and therefore cannot cross the separatrix on its own. This explains cases where a neuron in Class I is pushed toward the boundary but doesn't cross the separatrix (as in Fig3, answering to your comment above): the modulator in this case mostly changes *gL*, with too little effect on *a* or *tau_w*.
- To shift from Class I to II, a neuromodulator must increase a (stronger subthreshold adaptation) and/or prolong *tau_w* enough that *a*tau_w* > *C_m*. Many common receptor pathways inhibit M-type/AHP currents or they speed their kinetics. Moreover, *tau_w* is limited by channel kinetics and second-messenger bandwidth, and *a* is capped by available conductance. So, pushing *a*tau_w* above *C_m* is biologically harder than pulling it below.
- We have added these observations in the Method and Discussion.
